# Transcriptomic and genomic structural variation analyses on grape cultivars reveal new insights into the genotype-dependent responses to water stress

**DOI:** 10.1038/s41598-019-39010-x

**Published:** 2019-02-26

**Authors:** C. R. Catacchio, F. Alagna, R. Perniola, C. Bergamini, S. Rotunno, F. M. Calabrese, P. Crupi, D. Antonacci, M. Ventura, M. F. Cardone

**Affiliations:** 1 Consiglio per la ricerca in agricoltura e l’analisi dell’economia agraria (CREA), Centro di ricerca Viticoltura ed Enologia, Turi (BA), Italy; 2 Dipartimento di Biologia, Università degli Studi di Bari “Aldo Moro”, Bari, Italy; 3 Present Address: ENEA, Agenzia nazionale per le nuove tecnologie, l’energia e lo sviluppo economico sostenibile, Centro Ricerche Trisaia, Rotondella (MT), Italy

## Abstract

Grapevine (*Vitis vinifera* L.) is importantly cultivated worldwide for table grape and wine production. Its cultivation requires irrigation supply, especially in arid and semiarid areas. Water deficiency can affect berry and wine quality mostly depending on the extent of plant perceived stress, which is a cultivar-specific trait. We tested the physiological and molecular responses to water deficiency of two table grape cultivars, Italia and Autumn royal, and we highlighted their different adaptation. Microarray analyses revealed that Autumn royal reacts involving only 29 genes, related to plant stress response and ABA/hormone signal transduction, to modulate the response to water deficit. Instead, cultivar Italia orchestrates a very broad response (we found 1037 differentially expressed genes) that modifies the cell wall organization, carbohydrate metabolism, response to reactive oxygen species, hormones and osmotic stress. For the first time, we integrated transcriptomic data with cultivar-specific genomics and found that ABA-perception and –signalling are key factors mediating the varietal-specific behaviour of the early response to drought. We were thus able to isolate candidate genes for the genotype-dependent response to drought. These insights will allow the identification of reliable plant stress indicators and the definition of sustainable cultivar-specific protocols for water management.

## Introduction

Grapevine (*Vitis vinifera* L.) is one of the most important horticultural crops with a worldwide distribution. Vine development and grape ripening are sensitive to environmental factors, and climate change can affect production yield, grape composition, phenology and consequently the suitability for cultivation in whole territories. Among the parameters linked to climate change, there is the availability of water, which becomes increasingly limited and affects the quantity and quality of the production all over the world. Many vineyards, especially those devoted to table grape production, are located in areas, such as Mediterranean regions, California, Chile, and many others, often affected by severe water deficit. The cycle for table grape production involves the spring, summer and autumn, and is characterized by a high demand for water. This leads to an enormous consumption of freshwater resources. Thus, in order to promote a more sustainable viticulture, a reduction of water use has become essential. This is the challenge driving the recent researches to identify tolerant varieties with a better adaptation to water deficit.

*Vitis vinifera* L. has been described as relatively tolerant to water deficit, and regulated deficit irrigation has been advantageously used in wine-production as it induces an increase in total phenolic and anthocyanin content in fruits, which indeed influences the “sensory” characteristics and quality of wines^1–3^. However, water deficit (WD) negatively affects important aspects of table grape production, such as yield, berry size, firmness. Thus, irrigation remains fundamental to overcome water limitations.

At the physiological level, stomata closure, in response to water status declines, is one of the first responses to water deficit, in order to prevent the hydraulic failure^4^. Many scientists described variation in stomata control and proposed a physiological classification of plants as isohydric or anisohydric. Isohydric species can maintain a constant midday leaf water potential (Ψ_leaf_), by closing their stomata, regardless of soil water availability; whereas anisohydric species maintain higher stomatal aperture to optimize photosynthetic activity, but their Ψ_leaf_ significantly declines as soil water deficit increases. Ψ_leaf_ is typically lower in water stressed compared to well-watered plants, however, a clear variation from iso- to anysohydric behaviours has been observed between different varieties^5^. This behaviour is genetically controlled and quantitative trait loci (QTLs) controlling the maintenance of Ψ_leaf_ under moderate water deficit have been identified^6^. However, this framework is still debated^4^ considering that grapevine cultivars can exhibit, for instance, both near iso- or anisohydric behaviours depending on the environmental conditions^7,8^. In particular, a key role in the determination of the degree of iso/anysohydricity is played by the hydraulic properties of the soil^9,10^ and by the rootstock^11^. All these findings suggest that a strict division of grapevine varieties into isohydric and anisohydric categories is not possible.

At the molecular level, the phytohormone abscisic acid (ABA) plays a key role in mediating the stomatal responsiveness to WD. Indeed, the signal transduction cascade triggered by ABA, and involving ABA-induced gene expression, eventually leads to stomatal closure and water retention^12,13^.

Genome wide, large-scale expression analyses, and metabolomic studies have recently revealed new clues about the molecular basis of water stress responses in grapevine^8,14–20^. Mostly, these studies investigated the effect of water stress on quality traits important for the production of high-quality wines. Indeed, many scientists highlighted that WD induces many changes in secondary metabolism and revealed the importance of genes controlling stress-related signals cascades, such as those involved in membrane integrity, in water and ion uptakes, or related to reactive oxygen species (ROS) responses and osmotic protection. Notably, recent researches established the genotypic-specific response of grapevine to drought^8^. Inter-varietal differences and a dynamic physiological response to water availability have been described, thus revealing a different adaptation of grapevine varieties to the environmental conditions and a different ability to respond to water stress^4^. Comparative studies have investigated the relationship of transcriptomics, metabolomics and physiology in response to water stress^8,18^. However, studies integrating high throughput genomic data for the identification of the genetic traits responsible for the genotypic-specific response of grapevine to water deficit stress are still not available. These studies could help in the identification of traits conferring drought tolerance and the selection of varieties adapted to limited water availability.

The recent availability of plant genomic data and modern high throughput sequencing technologies have demonstrated that phenotypic variation in plant populations reflects the genetic diversity existing both at interspecific and intraspecific levels. High levels of structural variants have been found distributed throughout many plant genomes contributing to the phenotypic diversity^21–27^. Discovery and characterization of all forms of these genetic variations are crucial to reach a comprehensive understanding of the genetic basis of phenotypic differences. Recently, we created the first comprehensive map of genomic variations in grape genome and we demonstrated that grape genome is highly dynamic and subjected to structural alterations. This reveals the importance of structural variations in shaping the grapevine genomes^19,28^. Moreover, we highlighted that many gene families involved in stress response are affected by structural variations^19^.

In the present paper, we combined physiological, transcriptomic and, for the first time, also genomic data to study the grapevine response to water deficit in two table grape varieties, Italia (*It*) and Autumn royal (*AR*), cultivated in the South of Italy, which is one of the major production areas for table grape, in the Mediterranean basin, often affected by drought. The chosen cultivars showed, in preliminary assays, different tolerance to water deficit. They were tested directly on the field in order to evaluate their adaptation to growing conditions with reduced irrigation. Transcriptomic assays on apexes under WD and at full irrigation (FI) conditions revealed a great divergence in the response of these two cultivars, thus highlighting genotype-specific responses. More, by investigating NGS data, we were able to identify genomic variants putatively associated with the different ability to respond to water deficit.

## Results

### Irrigation treatments and plant physiological parameters

Plants of *AR* and *It* were subjected to two different irrigation treatments from fruit set until the harvest: control FI and WD corresponding to 100% and 60% of the net irrigation requirements, respectively. For the *It* cultivar, an additional point of over-irrigation (OI) was tested, corresponding to an increment of 50% of water supply with respect to FI.

**Figure 1:**
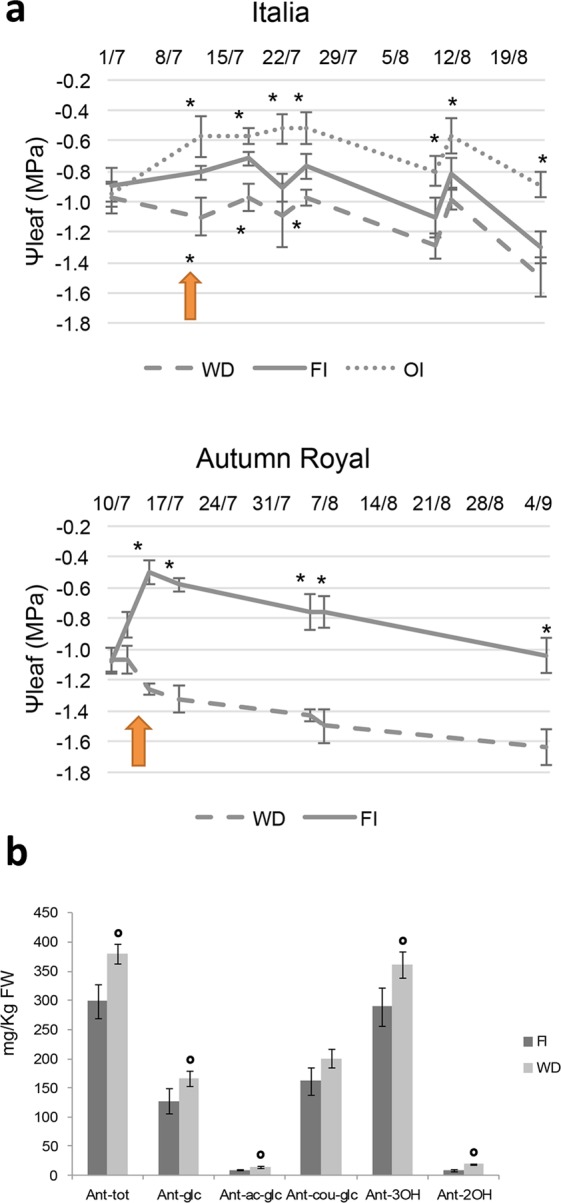
Effect of water deficit on physiological and qualitative parameters. (**a,b**) Evolution of leaf water potential (Yleaf) in Italia and Autumn royal during the entire seasonal irrigation. Arrows indicate sampling date for gene expression assays. Sampling was performed when Yleaf revealed water deficit stress. WD: water deficit; FI: full irrigation; OI: over-irrigation. Data are means ± S.D. *denotes significant (*p* < 0.01) difference from FI condition, as assessed by t test. (**c,d**) Anthocyanins content of Autumn royal table grapes experimenting two different irrigation treatments (FI vs WD). Values are expressed as mg/Kg of fresh weight (FW). FI: full irrigation; WD: water deficit. Data are means ± S.E. n = 3. °denotes significant (*p* < 0.05) difference.

The starting ψ_leaf_ value (around −1,0 MPa) corresponded to that found in grapevines from a region characterized by Mediterranean climate^29^. Irrigation treatment clearly affected vine water status as shown by the seasonal evolution of ψ_leaf_ in the two cultivars (Fig. 1a). During the first irrigation cycle, similar ψ_leaf_ values, ranging from approximately −0.8 to −1.0 MPa, were recorded for the vines treated with a deficit of irrigation. During the successive irrigation cycles, water supplies was reduced and a decrease of the ψ_leaf_ of vines under reduced irrigation was detected, reaching minimum values of approximately −1.6 and −1.5 MPa for *AR* and *It*, respectively. Noteworthy, ψ_leaf_ decreased more rapidly in *AR* than in *It* and at the end of the treatment differences in ψ_leaf_ were higher in *AR*.

Excess of irrigation is often practised for table grape production; therefore, a condition of OI was also tested for cultivar *It*. As expected, the ψ_leaf_ showed higher levels than in the FI (Fig. 1a). For leaf gas exchange, all parameters showed a decline in the thesis with reduced water intake and an increase of the intrinsic water use efficiency (Supplementary Note §1.1), thus confirming the stress status in both cultivars. The comparison of the physiological overall data (Supplementary Note §1.1) suggested that WD treatment resulted in better intrinsic water use efficiency in *AR* with respect to *It*.

### Effect of different irrigation treatments on berry quality and production

We investigated the influence of the applied water stress on the productivity of the two cultivars and fruit quality at harvest time (Supplementary Note §1.2). In agreement with previously reported data^30^, we observed a negative influence of WD on cluster numbers and bunch weight, which resulted in a lower production per vine in both cultivars. Moreover, we also measured a reduction of berry size. Quality parameters such as sugar content, Ph and total acidity did not show significant variations, despite a global reduction of the sugar production per vine.

As previously reported^31^, *AR* is a black table grape variety mainly characterized by high content of tri-hydroxylated anthocyanins (Ant-3OH), even though consistent levels of di-hydroxylated anthocyanins (Ant-2OH) are also present. We found a significant increment of total anthocyanins content upon the WD application compared to FI (Fig. 1b). This trend was particularly evident either for Ant-3OH and for Ant-2OH anthocyanins (with an increment of 1.4 and 2.5 folds, respectively) in both glucosyl and acetyl-glucosyl forms. Conversely, we did not register any difference in the case of anthocyanidins-3O-coumaryl-glucosides.

### Effect of water deficit on grapevine gene expression

In order to study the effect on grapevine gene expression of a reduction of irrigation supply in field condition, leaf apexes of plants of *AR* and *It* subjected to FI and WD conditions were collected when WD plants showed water deficit stress, two days after the first cycle of the differentiated irrigation treatment. We used these samples for microarray analyses.

**Figure 2:**
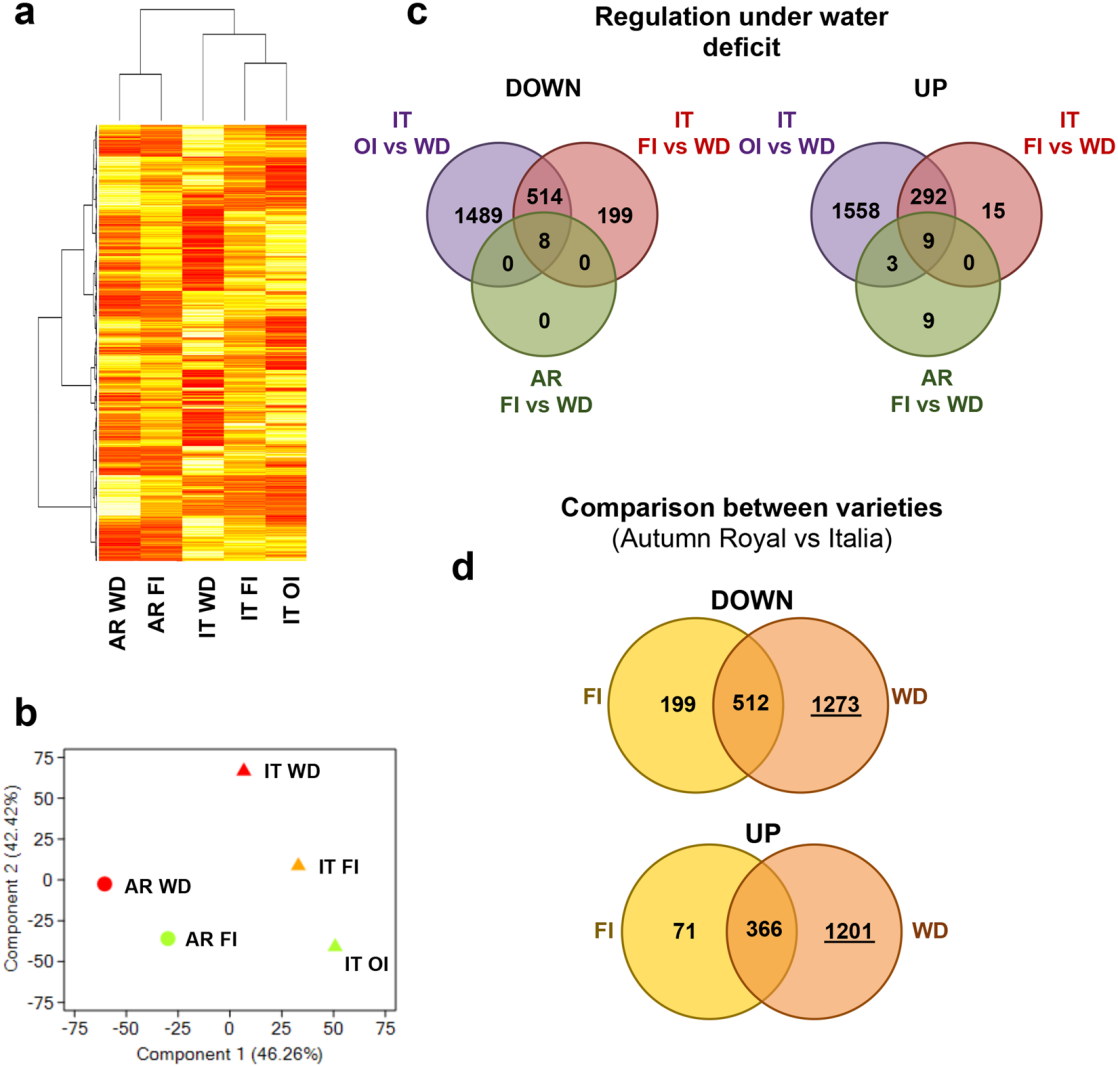
Identification of genes differentially expressed under water deficit in table grape varieties Italia and Autumn royal. (**a**) Hierarchical Clustering Analysis (HCA) of DEGs. Colors indicate transcriptional activation (red) or repression (yellow). The columns and rows represent samples and genes, respectively, that were grouped based on their expression profile. (**b**) Principal Component Analysis (PCA) depicting global gene expression profile of *AR* and *It* at different water conditions. The analysis highlights that variation between cultivars is higher than between conditions. (**c**) Venn diagrams show down- and up-regulated genes between different water conditions. The comparison between OI and FI in *It* does not reveal any significant DEGs. Italia significantly modulated a total of 1037 genes (316 up- and 721 down-regulated), whereas *AR*, despite an overall variation of expression profile, showed only 29 DEGs between FI and WD (21 up- and 8 down-regulated). Noteworthy, 20 of these genes were common to *It* responses, whereas, nine genes were modulated exclusively in this cultivar. (**d**) Venn diagrams show down- and up-regulated genes in *AR* compared to *It*. The higher variation of gene expression between cultivars is depicted at WD (the number of DEG is underlined). AR: Autumn royal; IT: Italia; WD: water deficit; FI: full irrigation; OI: over-irrigation.

Transcripts showing a fold change ≥2 with *p* < 0.05 were considered as differentially expressed genes (DEGs): (a) in each cultivar under water deficit (Supplementary Dataset 1); (b) between the two cultivars at both FI and WD conditions (Supplementary Dataset 2); (c) between OI and WD in *It* (Supplementary Dataset 3). Hierarchical clustering and Principal Component Analysis (Fig. 2) of gene expression data showed a wide variation of gene expression between both water conditions and cultivars, evidencing that the variation between genotypes was higher than between conditions. The comparisons between FI and WD conditions indicated a cultivar-specific response to water deficit (Fig. 2).

Differences between cultivars strongly increased at WD compared to FI, as most of the DEGs between the two cultivars at WD are specific to this condition (2474 out of 3352) (Fig. 2).

With regard to the effects of an excess of water on gene expression profile, the comparison between OI and FI in *It* did not reveal any significant DEGs, whereas we identified a total of 3873 DEGs (1862 up- and 2011 down-regulated) between OI and WD (Supplementary Note §1.3).

We further validated the observed trend on apexes of seven DEGs by real time analyses on leaf and tendril of *It* at OI, FI and WD conditions and on leaf of *AR* at FI and WD conditions. The analysis confirmed the expression trend observed by microarray analysis on apexes (Supplementary Note §1.4).

The MapMan analysis provided an overview of the metabolic pathways and regulatory networks affected by water deficit in the two studied cultivars (Supplementary Note §1.5).

**Figure 3:**
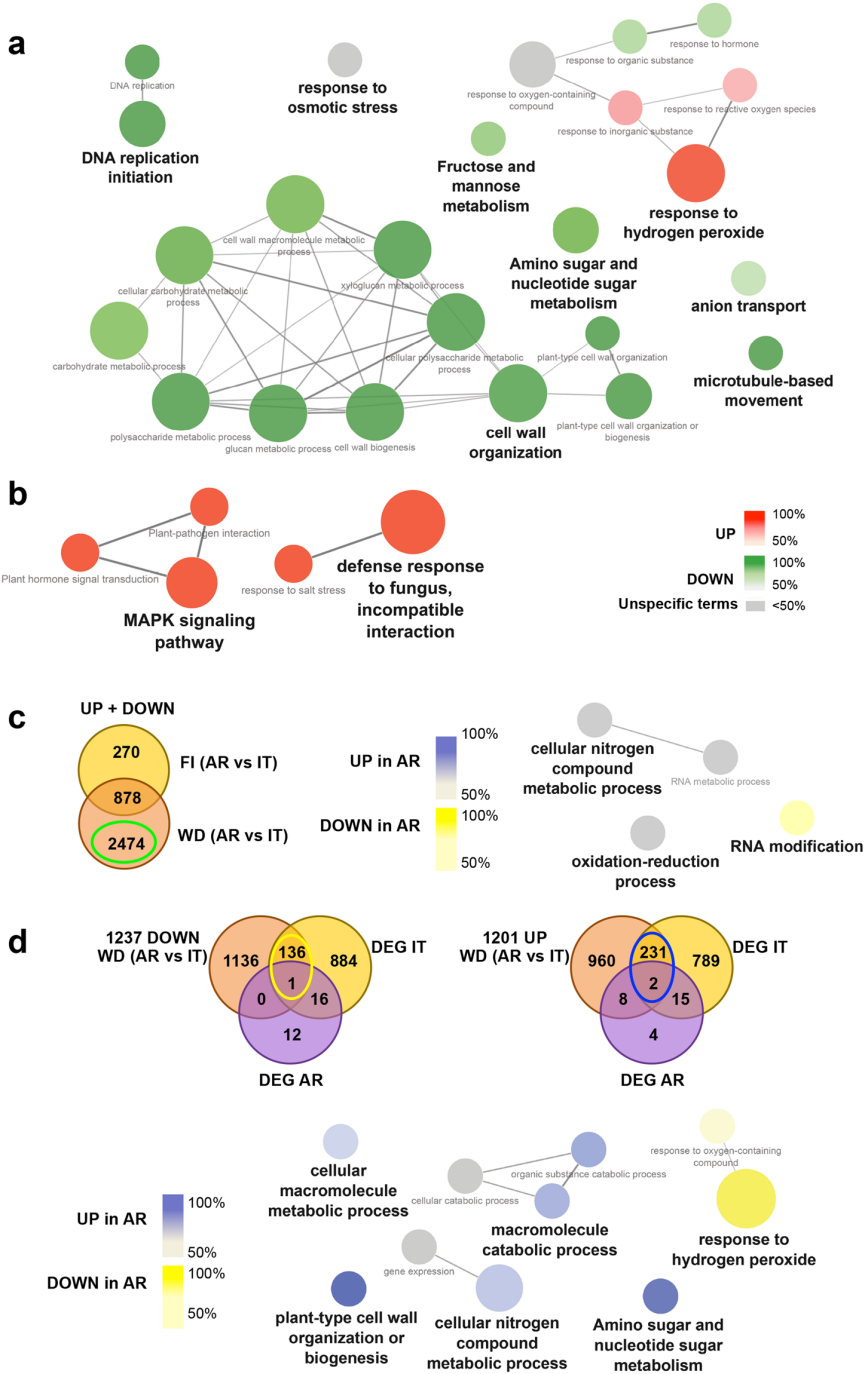
Network analysis of genes differentially expressed under water deficit in table grape cultivars Italia and Autumn royal. (**a,b**) Network of genes differentially expressed under WD in cultivars *It* and *AR*, respectively. For cv. *It* only terms containing at least three genes were shown, this restriction was not applied for cv. *AR*. Nodes with up- or down-regulated genes are shown in red or green, respectively. (**c,d**) Network of genes differentially expressed between cvs. *It* and *AR* at water deficit condition (WD) and regulated under water stress, as indicated by Venn Diagrams. Yellow- and blue-circled numbers represent, respectively, selected down- and up-regulated genes between *AR* vs *It*. Nodes with up- or down-regulated genes (*AR* was used as reference) are shown in blue or yellow, respectively. Data are visualized as clusters distribution network (Cytoscape, ClueGO App). Only significant (*p* < 0,005) terms belonging to GO biological process and Kegg ontologies were shown. The node size is proportional to the term significance. The colour gradient shows the proportion of up- and down-regulated genes associated with the term. Equal proportions of both clusters are represented in gray. AR: Autumn royal; IT: Italia; WD: water deficit; DEG: differentially expressed genes.

We used the Cytoscape plug-in ClueGo to analyze the DEGs in order to identify functionally grouped annotation networks (Fig. 3). Only significant (*p* < 0.005 or *p* < 0.001) terms belonging to GO biological process and Kegg ontologies were considered for the network analysis in order to select the most significant biological process and pathways affected by water deficit.

#### Cultivar-specific response to water deficiency

Networking analyses of the DEGs between FI and WD conditions revealed a cultivar-specific response to WD. In particular, *It* showed a predominant down-regulation of genes involved in the primary metabolism (DNA replication, carbohydrate metabolism, and cell wall organization), coupled with a modulation (up- or down-regulation) of genes involved in the responses to stress (osmotic stress, response to oxygen containing compounds, inorganic substances, and hormones) (Fig. 3). In contrast, in *AR*, the modulation of gene expression under WD was limited to a very little number of genes but most of them are specifically involved in the plant response to stress conditions (Fig. 3).

In order to increase the comprehension of the main functional networks affecting the different response to water deficit of *It* compared to *AR*, the total number of DEGs between *AR* and *It* at WD condition have been analysed. Despite the large number of genes studied (a total of 2474 genes: 1201 up- and 1273 down-regulated in *AR* compared to *It*), the analysis showed that most of them do not group in any significant network. Only a few significant pathways of DEGs between the two cultivars were identified: nitrogen metabolism, oxido-reduction process, RNA metabolism and modifications (Fig. 3).

To select, among the DEGs between cultivars, those specifically modulated in responses to WD, we intersected them with the DEGs between FI and WD conditions (Fig. 3). The genes common to these two groups (137 down- and 233 up-regulated in *AR* compared to *It*) have been used for gene networking analysis. The results indicated that *AR* at WD showed a predominant up-regulation of genes involved in the following biological processes: cell wall organization, macromolecule metabolic process, amino- and nucleotide-sugar metabolism and a predominant down-regulation of genes involved in the response to oxygen containing compounds. These results agree with the modulation of genes belonging to these pathways in *It* under WD. Interestingly, the analysis indicated nitrogen metabolism as a significant pathway modulated between the two varieties, confirming its importance in determining the different response to WD of the two cultivars.

#### Main biological processes characterizing grapevine response to water deficit

Considering the overall results of network analyses, a complete list of DEGs belonging to the main pathways putatively involved in the modulation of grapevine defence responses to water deficit and their cultivar-specificity has been reported (Supplementary Dataset 4).

According to the important role of phytohormones in the regulation of plant stress response we found, respectively, 45 down- and 34 up-regulated genes in response to WD in cultivar *It*, whereas, four up- and one down-regulated genes were identified in *AR* (Supplementary Note §1.6). ABA, auxin and ethylene responsive genes were predominant among the DEGs. Three genes characterize the response of *AR* (up-regulated exclusively in this cultivar) and encode for pathogenesis-related proteins putatively associated to salicylic acid signalling. According to this, we found ABA response elements (ABRE) and ABRE-related motifs in 42 hormone-responsive DEGs (Supplementary Note §1.6). Interestingly, differences between *AR* and *It* were identified. In particular, ABRE elements were found in the promoter region of *DREB1A* (VIT_16s0100g00380) and two *ERF5* encoding genes (VIT_16s0013g01060, VIT_16s0013g01050) in cultivar *It*, but the same regions in *AR* do not contain these elements.

As expected, under WD condition, we identified 22 up- and 23 down-regulated genes involved in osmotic and water stress response in *It* (Supplementary Dataset 4). Among them, six genes encode for dehydration responsive proteins (*RD22*, *RD26*, *XERICO, DRS1)* and transcription factors regulated by ABA (*DREB1A, MYB102*).

Noteworthy, two osmotin-like genes *OSM34* generally associated to drought tolerance in other species resulted up- (VIT_02s0025g04340) and down-regulated (VIT_02s0025g04230). In contrast to the large number of genes differentially expressed in It, only two genes involved in osmotic stress response resulted differentially expressed in *AR*, both up-regulated: b-glucanase (VIT_205s0077g01150) and OSM34 (VIT_02s0025g04340), similarly to *It*.

Modulation of 41 genes involved in the response to oxygen-containing compounds was observed in *It* (Supplementary Dataset 4). They include genes involved in the generation of reactive oxygen species (ROS) as peroxidases, in the plant defence from ROS (as catalases, ascorbate peroxidases) and in the cell redox homeostasis (as thioredoxins). Only one gene involved in these pathways was differentially expressed (up-regulated) in *AR*: the peroxidase 5-like (*RCI3*: VIT_14s0060g00520).

**Figure 4:**
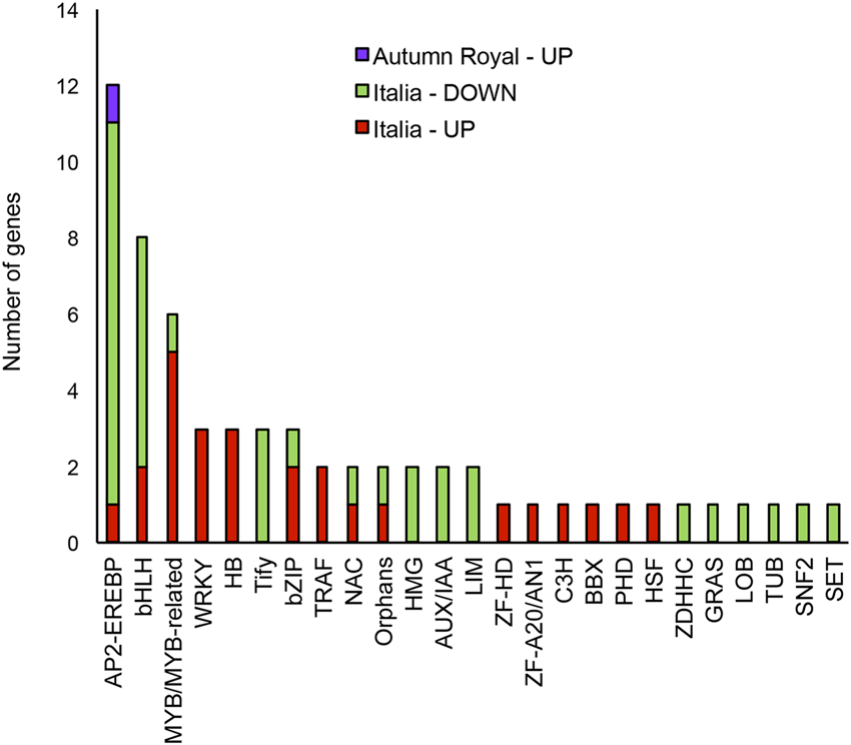
Transcription factors families differentially expressed under water deficit. The proportion of genes up-regulated in *AR*, down- and up-regulated in *It* is indicated in purple, green and red, respectively. AP2-EREB: APETALA2 and ethylene responsive element binding proteins characterized by AP2 DNA-binding domain; bHLH: basic helix-loop-helix family of proteins; MYB/MYB related: proteins containing Myb DNA-binding domain; WRKY: WRKY domain transcription factors; HB: Transcription factors containing homeobox KNOX1 KNOX2 domains; Tify: proteins characterized by Tify domain; bZIP: basic region/leucine zipper motif (bZIP) transcription factors; TRAF: proteins containing BTB domain; NAC: plant-specific transcription factors possessing NAM domain; Orphans: Orphans family transcription factors; HMG: HMG-box DNA-binding transcriptional regulators; AUX/IAA: proteins, containing AUX/IAA domain, that repress expression of primary/early auxin response genes; LIM: LIM domain proteins; ZF-HD: zinc finger homeodomain proteins; ZF-A20/AN1: Zinc finger A20/AN1-type domain proteins; C3H: Cys3His zinc finger domain proteins; BBX: zinc finger b-box domain proteins; PHD: PHD domain containing proteins; HSF: heat shock transcription factors containing a HSF DNA-binding domain; ZDHHC = DHHC zinc finger domain containing proteins; GRAS: GRAS domain containing proteins; LOB: plant-specific protein family containing DUF260 domain; TUB: TUBBY-like proteins containing a TUB domain.

In addition, a complete list of the transcription factors (TF) differentially expressed under WD, has been also reported (Supplementary Dataset 4). According to the evident differences in the response to WD between *AR* and *It*, we found 61 TF differentially expressed (35 down-regulated, 26 up-regulated) in *It* versus only one TF differentially expressed (up-regulated) in *AR*. Most of TFs belong to AP2-EREB, bHLH and MYB families (Fig. 4).

### Inter-cultivar comparison of genomic data

In order to find genotype-specific differences putatively associated to the observed different response to WD, we looked for *AR* and *It* specific genomic variations among previously published data^19^ searching for polymorphic genes between the analysed cultivars involved in abiotic stress. More in detail, we focused our attention on copy number variant (CNV) regions and single nucleotide variants (SNVs) affecting gene transcription. By looking at the annotated copy number for each cultivar, we identified 1249 subregions whose *AR* and *It* copy number difference was higher than 0.5 (Table 1, Supplementary Dataset 5). Notably, this indicates that about 5% of the grape genome is variable between the two cultivars. Interestingly, the extracted subregions were found to overlap with 1499 genes. Interestingly, four regions showed very high CN differences between *AR* and *It* (>20); these regions had already been described as hyper-duplicated regions by Cardone *et al*.^19^. Two of these contain genes belonging to NADH dehydrogenases family and for both of them *AR* showed the higher CN with respect to *It*. Genes of which the copy number difference was higher than 5 (147 genes corresponding to 111 regions), were mainly found to belong to gene families involved in stress responses (*e.g*. NBS-LRR and Ankyrin domain proteins) or to relate to transposable elements.

**Table 1:** CNVs and SNPs identified comparing *AR* and *It* high-throughput sequencing data.

Genomic variants	CNVs	SNVs
Total polymorphisms	1249 regions (1499 genes)	2283 (1807 genes)
Overlapping with genes involved in functional categories of interest^a^	26 (27 genes)	49 (41 genes)
Overlapping with genes differentially expressed between *AR* and *It*	159 (159 genes)	336 (295 genes)

^a^Genomic variants overlapping with genes belonging to both abiotic stress response and hormone signalling functional categories.

While studying the SNVs between the two cultivars, instead, we found 2283 events functionally affecting 1807 genes (*i.e*. causing a gain or loss of function) (Table 1, Supplementary Dataset 7). The functional categories of these genes are mainly represented by: primary metabolism (908 SNVs), response to stress (including ankyrin, NBS-LRR and response to stimulus) (205 SNVs), signalling (159 SNVs), transport (111 SNVs), secondary metabolism (79 SNVs), cellular processes (78 SNVs) and transcription factors (64 SNVs).

Among the found variations, 876 SNVs were found exclusively in *AR* (*i.e*. showing a homozygous reference genotype in *It*: 0/0), while 1025 SNVs were found exclusively in *It* (*i.e*. showing a homozygous reference genotype in *AR*: 0/0). Among these, 133 and 101 SNVs were homozygous (genotype 1/1) in *AR* and in *It*, respectively.

We deeply studied the CNVs and SNVs containing genes involved in pathways of relevance in the response to abiotic stresses and drought in particular (26 CNVs and 49 SNVs) (Supplementary Dataset 6). Among these, *HVA22A* and *RD22* (related to ABA signalling and water stress response) were found more duplicated in *It* compared to *AR*. Noteworthy, one *RD22* (VIT_04s0008g04120) and two *HVA22A* (VIT_03s0097g00470 and VIT_03s0038g01650) genes also showed cultivar specific SNVs causing stop codon gains.

In order to gain insights into the genetic bases of cultivar-specific response to water deficit, we compared the so highlighted genomic differences with the DEGs found in our comparisons (Supplementary Dataset 8). We found 158 DEGs showing differences in CN. Looking at the functional annotation, we found enrichment of the following categories: primary metabolism (37 DEGs), stress response (including NBS-LRR and response to stimulus) (32 DEGs), secondary metabolism (terpenoid and phenylpropanoid metabolisms) (14 DEGs), transport (13 DEGs), signalling pathway (12 DEGs). 54% revealed a direct correlation between CNV and expression level (*i.e*.: the higher CN corresponded to UP regulation). A selection of the genes putatively involved in the cultivar-specific response to water deficit has been reported in Table 2. Interestingly, the different CN of *HVA22* and *RD22* might explain their differential expression. Of note, a lineage-specific evolution of the *RD22* family has been described with the biggest expansion in grapevine^32^. We found 18 paralogues in the reference genome and identified CNVs between *AR* and *It* in ten of them^19^. VIT_04s0008g04140 and VIT_04s0008g04150 showed also down regulation in *AR* with respect to *It* (Table 2).

**Table 2:** CNVs occurring in genes differentially expressed between *AR* and *It* selected as candidate genes for grapevine response to water deficit stress.

Copy number		Unique ID	Regulation between cvs	Functional annotation	Network
*AR*	*It*				
16.91	22.89	VIT_03s0132g00070	DOWN in *AR* at WD and FI	HVA22A	ABA and drought response
11.41	15.30	VIT_03s0132g00080	DOWN in *AR* at WD and FI	HVA22A	
1.68	2.31	VIT_04s0008g04140	DOWN in *AR* at WD	RD22	
1.18	2.81	VIT_04s0008g04150	DOWN in *AR* at WD and FI	RD22	
13.06	4.58	VIT_09s0002g07820	UP in *AR* at WD	Quinone oxidoreductase	Electron transport (Respiratory-chain phosphorylation)
11.30	3.95	VIT_09s0002g07820	UP in *AR* at WD	Quinone oxidoreductase	
8.18	8.94	VIT_13s0047g00080	DOWN in *AR* at FI	ZIFL1 (Zinc induced facilitator 1)	H+ Transport
10.22	11.19	VIT_13s0047g00080	DOWN in *AR* at FI	ZIFL1 (Zinc induced facilitator 1)	
4.68	5.28	VIT_12s0059g00050	DOWN in *AR* at WD	Ankyrin repeat	Transport
2.53	3.10	VIT_14s0128g00120	DOWN in *AR* at WD	Ring-H2 finger protein VVL3B	Regulation (zinc finger C_3_HC_4_ family)
2.51	5.14	VIT_02s0025g01900	DOWN in *AR* at WD	Cellulose synthase CSLG3	Cell wall organization and biogenesis
2.20	1.14	VIT_16s0013g01680	UP in *AR* at WD	L-asparaginase 3 precursor	Nitrogen metabolism and assimilation
13.89	8.87	VIT_16s0013g01680	UP in *AR* at WD	L-asparaginase 3 precursor	
19.32	13.22	VIT_16s0013g01680	UP in *AR* at WD	L-asparaginase 3 precursor	
5.98	3.39	VIT_03s0063g01220 VIT_03s0063g01250	UP in *AR* at WD	Nodulin 1 A. senescence-associated	
3.30	1.74	VIT_16s0100g00090*	UP in *AR* at WD	Cationic peroxidase	Aromatic amino acid metabolism (Phenylalanine metabolism)
4.76	10.09	VIT_18s0001g04690	DOWN in *AR* at WD	Sesquiterpene cyclase	Terpenoid biosynthesis
6.93	23.65	VIT_18s0001g04690	DOWN in *AR* at WD	Sesquiterpene cyclase	
10.12	3.14	VIT_18s0001g06160	UP in *AR* at WD	TIR-NBS-LRR-TIR disease resistance protein	Plant-pathogen interactions
11.93	5.03	VIT_12s0034g01660	UP in *AR* at WD	R protein MLA10	
2.80	6.95	VIT_13s0064g01850	DOWN in *AR* at WD	R protein MLA10	
5.58	8.66	VIT_07s0141g01030	DOWN in *AR* at WD	R protein MLA10	
4.04	6.35	VIT_00s0222g00080	DOWN in *AR* at WD	CC-NBS-LRR class	

Likewise, we compared SNVs with DEGs and we found 336 SNVs affecting the function of 295 DEGs. Among these, to identify candidate genes responsible for the observed genotype-dependent response to water stress, we specifically looked at SNVs corresponding to inter cultivar DEGs and we found 22 genes (Table 3).

**Table 3:** SNPs occurring in genes differentially expressed between *AR* and *It* selected as candidate genes for grapevine response to water deficit stress.

SNP	Cultivar	Unique ID	Regulation between cvs	Functional annotation	Network
chr3_9101157_A/T	*AR* het	VIT_10s0003g04500	UP in AR at WD	Ankyrin	Ankyrin repeat protein
chr3_10715509_C/A	*AR* het				
chr3_10713269_G/A	*AR* homo/It het				
chr4_17564196_A/T	*AR* het	VIT_11s0037g01350	UP in AR at WD	Ankyrin	
chr5_18992761_C/T	*AR* het	VIT_13s0106g00060	DOWN in AR at WD	Ankyrin repeat	
chr8_17864017_C/T	*It* het	VIT_09s0002g01900	UP in AR at WD	APM1 (Aminopeptidase M1)	Auxin Transport
chr8_17864127_C/A	*It* het	VIT_05s0062g00720	DOWN in AR at WD	UDP-glucoronosyl/UDP-glucosyl transferase UGT75C1	Flavonoid biosynthesis
chr8_8569440_T/G	*AR* het	VIT_00s0207g00160	UP in AR at WD	Pathogenesis-related protein 1 precursor (PRP 1)	JA and SA signalling
chr9_1663113_G/A	*AR* het	VIT_03s0088g00890	UP in AR at WD	Pathogenesis related protein 1 precursor [*Vitis vinifera*]	
chr9_8958872_C/T	*It* het	VIT_17s0000g07400	UP in AR at WD	EDS1 (Enhanced disease susceptibility 1)	
chr10_7866464_T/A	*It* het	VIT_17s0000g06810	DOWN in AR at WD	Myb family transcription factor/ELM2 domain-containing	MYB Transcription factor
chr10_7866668_C/A	*It* het	VIT_08s0058g00070	DOWN in AR at WD	Disease resistance protein SlVe2 precursor	NBS-LRR superfamily
chr10_7866709_C/T	*AR* het	VIT_09s0002g08270	DOWN in AR at WD	Leucine-rich repeat	
chr11_10805976_C/T	*AR* homo	VIT_13s0064g00470	DOWN in AR at WD	Disease resistance protein (NBS-LRR class) RGH1	
chr13_22086745_G/C	*It* het	VIT_16s0022g00240	UP in AR at WD	EIX receptor 2	
chr13_9391005_C/T	*AR* homo/It het	VIT_18s0041g00160	DOWN in AR at WD	Disease resistance protein (NBS class)	
chr14_26132777_G/A	*AR* het	VIT_19s0027g00830	DOWN in AR at WD	Disease resistance protein (CC-NBS-LRR class)	
chr16_11039713_C/T	*It* het	VIT_14s0171g00300	DOWN in AR at WD	4-coumarate-CoA ligase	Phenylpropanoid biosynthesis
chr17_7466883_T/A	*AR* het	VIT_08s0007g03880	DOWN in AR at WD	Zinc finger (C_2_H_2_ type) family	Regulation (zinc finger C_2_H_2_ family)
chr17_8334525_A/G	*AR* het				
chr18_r_3865601_C/T	*It* het	VIT_04s0023g01130	DOWN in AR at WD	E3 ubiquitin-protein ligase RNF5	Regulation (zinc finger C_3_HC_4_ family)
chr18_24517362_C/T	*It* het	VIT_00s0271g00060	UP in AR at WD	Linalool synthase	Terpenoid biosynthesis
chr19_5156975_A/C	*It* het	VIT_18s0001g04480	UP in AR at WD	Germacrene-D synthase	
chr19_20163598_A/G	*AR* het	VIT_19s0014g04810	DOWN in AR at WD	Vetispiradiene synthase	
chrUn_12494276_T/A	*It* het	VIT_03s0097g00470	UP in AR at WD	ATHVA22A (Arabidopsis thaliana HVA22 homologue A)	ABA signalling

## Discussion

Water deficit represents the main environmental constraint for growth in grapevine. In response to drought, different cultivars adopt different strategies to limit the effect of water deficit, although the genetic and physiological origins of these differences are still debated^8,18,33,34^.

In the present work, we combined physiological studies with transcriptomics and genomics in order to investigate the different ability of two table grape varieties to respond to water stress and highlighted new clues about the genetic bases of these differences. Most of the previous literature data are based on experiments under controlled conditions, and they are mostly focused on wine grape varieties^35^. We, instead, tested the effects of a reduced irrigation directly on the field in order to investigate how the mentioned different ability reflects an adaptation of the cultivars to the growing conditions.

We demonstrated a negative effect of WD on some important traits for table grape production such as bunch weight and berry size, confirming previous results^30^.

Besides water stress does not induce a constant response in flavonols and flavanols^36,37^, anthocyanins exert homogeneous behaviour increasing under WD^35,38,39^. For this reason, we decided to focus our attention on the different classes of anthocyanins in *AR* (Fig. 1b) and we demonstrated that the WD affects positively anthocyanin content, especially on anthocyanins-3O-glucosides and 3O-acetyl-glucosides. Moreover, we showed that the relative amount of ant-2OH increased more than the ant-3OH one (2.1 vs 1.2 folds, respectively) (Fig. 1b). Instead, previous studies have described a shift in biosynthesis toward a higher proportion of 3′,4′,5′-trihydroxylated compared to 3′,4′-dihydroxylated anthocyanins through the up-regulation of flavonoid-3′,5′-hydroxylase^37,39,40^.

It has lately been annotated, however, that also phenylpropanoids and terpenoid pathways can take part in the berry response to WD in non-pigmented berries, suggesting that an overproduction of monoterpenes is part of the fruit response to drought^20^. We accordingly found structural variations between *It* and *AR* in DEGs belonging to terpenoid and phenylpropanoid gene families (Tables 2 and 3).

Recently, many comparative studies have addressed the topic of water stress response in grapevine at a molecular level, using different experimental approaches^8,14,16–18,20,41^. With respect to previous data, we focused our attention on the early response to water deficit. Besides differences in experimental settings, pedo-climatic and growing conditions, other than tissues analysed, our data confirmed that WD induces modulation in genes related to response to stimuli, response to abiotic stress, ABA response, protein and carbohydrate metabolisms, nitrogen metabolism, and ROS response, thus revealing the importance of such pathways in the response to water stress. Notably, both the analyzed varieties showed modulation of genes related to osmotic stress response and those related to the primary immune plant system such as the defence proteins (PR1). This finding demonstrated that the mechanisms acting in the primary response to both biotic and abiotic stresses are shared as already highlighted for other kind of stresses^18^.

However, comparing our data with those already available^42^ we found qualitative and quantitative differences in the genes specifically modulated in response to WD. Indeed, only 8–10% of the DEGs found in our experiments overlap with those recently selected as responsive genes in Montepulciano and Sangiovese^8^. This strongly supports the genotype-dependent response to WD.

In accordance with this, we found strong differences in the WD response between *AR* and *It*. *AR* showed a limited and specific response, involving the modulation of genes specifically related to plant defence mechanisms, including drought-responsive genes such as desiccation proteins.

The strong differences observed between *AR* and *It* under WD stress might also depend on a different timing of response between the two cultivars: *AR* could activate later a more extensive response to WD, similarly to what found for the anisohydric cultivar Sangiovese^8^. This suggests that the genotype-specific responses to WD need to be investigated at the early phases after WD.

This behaviour could probably reflect a better adaptation of *AR* to the WD conditions. Indeed, adaptation and resilience to water stress, such as the extremely limited response in the early phase found in *AR*, could be considered more advantageous. In this way, the plant could activate its defence responses more gradually - only if the WD condition is prolonged - and this could avoid investing much resources and energy if not strictly necessary.

In order to understand the molecular basis of such kind of genotypic - specific response, we deeply analyzed NGS data belonging to the studied cultivars and we identified structural variants in stress-related genes. Many genomic variations were also correlated to the expression differences, and thus putatively associated to the different genotypic-specific behaviour observed in response to WD. Genomic data obtained from different plant species have revealed that plant genomes are highly plastic as a result of different mechanisms such as genome duplication, segmental duplications, and transposable elements (TEs) mobility. Moreover, contrary to what was previously thought, it is now clear that the genetic plasticity is useful for the adaptation to a changing environment^43^.

Among the most polymorphic genes between *AR* and *It*, we found that the higher CN differences affected some well-known stress-related gene families, such as ankyrin repeat proteins belonging to the RING finger family, recently described as specifically related to drought response in *Arabidopsis thaliana*^44,45^. Indeed, the *Arabidopsis thaliana* ankyrin *DRA1*_(At4g03500) was identified as a negative regulator of drought tolerance^45^. Interestingly, there are 18 orthologs of this gene annotated in grape (Gene Tree EPlGT00940000163197, Ensembl Plants release 41) and 12 of these had shown a differential expression in response to WD between *AR* and *It* (Table 2, Supplementary Dataset 2). Of note, the gene VIT_12s0059g00050, down-regulated in *AR* in response to WD, showed a lower CN in *AR* with respect to *It*. Additionally, we also found five SNVs (of which four specifically in *AR*) causing a gain of a stop codon, in three genes coding for ankyrin repeat proteins (Table 3). These data, in agreement with what previously known about ankyrin proteins in *Arabidopsis thaliana*^44,45^, reveal the role of these proteins in the genotype-dependent response to drought. Stress conditions could have induced gene duplications and these events could create genome plasticity leading to a different ability to respond to the changing environment.

Antioxidant enzymes, metabolites, transcription regulators, and cross-talk with hormones prompted by abiotic stress conditions are crucial to ensure the right antioxidant homeostasis, achieving a positive balance between photosynthesis and respiration^33^. According to this, among the genes showing CNVs and SNPs directly related to the expression modulation in early stress responses we also found genes involved in photosynthesis, energetic metabolism, electron transport and ROS scavenging pathways as NADH dehydrogenases and quinone oxidoreductases (Supplementary Dataset 8).

Of note, we also found more than 200 TEs showing different CN in *AR* and *It*. It is well known that stresses induce activation of TEs in plants and that the resulting genome plasticity is fundamental to survive in adverse environments^46^. Rocheta and colleagues^18^ described differential expression of TEs in grapevine in a stress-specific manner, suggesting a role of TEs in grapevine stress response and adaptation response to abiotic stresses.

According to the importance of phytohormones in the regulation of plant responses and adaptation to abiotic and biotic stresses^47–50^, and the key role of ABA in the modulation of the complex hormonal network in response to WD^51,52^, we identified numerous differences between *AR* and *It* not only at transcriptomic but also at genomic level. Indeed, we revealed CNVs in 30 genes of phytohormone signalling and perception, most of them ABA-dependent (Supplementary Dataset 6). Our results confirmed that ABA-mediated perception and response might be the major responsible for the varietal-specific behaviour observed applying water stress.

As an example, higher CN in the gene *RD22*, in *It*, is also coupled with a significantly higher expression of these genes in cultivar *It* compared to *AR* (Table 2). The Responsive to Dehydration 22 (*RD22*) has been recently described as a link between ABA signalling and abiotic stress responses^32^ by maintaining cell integrity under stress conditions^53^. Notably, a big cluster of paralogous copies of *RD22* on chromosome 4 has been described^32^ and it is a hot spot for subsequent duplication/deletion events mediated by unequal crossing-overs, leading to CNV in this region^19^. According to Matus and colleagues, we found that different members of the grape *VvRD22* group present a different expression during the early response to water stress. In addition to this, we also found CNVs in this gene family between *AR* and *It*, thus supporting the role of *RD22* in ABA-mediated response in a genotype-dependent way.

Moreover, we found that the ethylene responsive factors (ERFs) resulted the most represented TF family differentially expressed under WD, counting 12/62 genes. Nine of them, possess ABA-responsive elements (ABRE or ABRE-related) in their promoters. Eight of these ABA-responsive ERFs resulted down-regulated in response to WD stress, whereas, one was up-regulated (Supplementary Dataset 4). This is in agreement with literature data on different plant species, showing that ERFs might regulate the drought stress responses in both directions: overexpression of some ERFs enhance the tolerance to water stress, whereas others could act as repressors in drought stress responses^54–58^.

Other important players in the regulation of ABA responses to WD include the type 2 C protein phosphatases as ABI1. These proteins regulate the sensitivity of guard cells K+ channels to this phytohormone, indeed *abi1* and *abi2* arabidopsis mutants are insensitive to ABA and unable to close stomata^59^. We showed that WD stress increases *ABI1* transcription only in *It*, however, it is not possible to depict the consequent effect on ABA regulation, considering the complex regulation occurring at this stage of ABA signalling.

ABF2, an ABRE-binding bZIP factor, is another component of ABA signalling likely involved in the adaptive processes to abiotic stresses as drought. Overexpression of this gene enhances resistance to water stress and induces stomatal closure^60^. Interestingly, our results indicate an up-regulation of *ABF2* in *It* in response to WD condition. The increase of its expression level might be responsible of the transcriptional activation of other ABA-responsive genes related to stress response. According to this hypothesis, we found ABRE motifs in the promoter of drought related genes as *RD22, ERF, DREB1, DDF2*. Interestingly, differences in the presence/absence of ABRE motifs were also found between *AR* and *It*, they might affect the ABA-mediated response of the two cultivars under WD.

**Figure 5:**
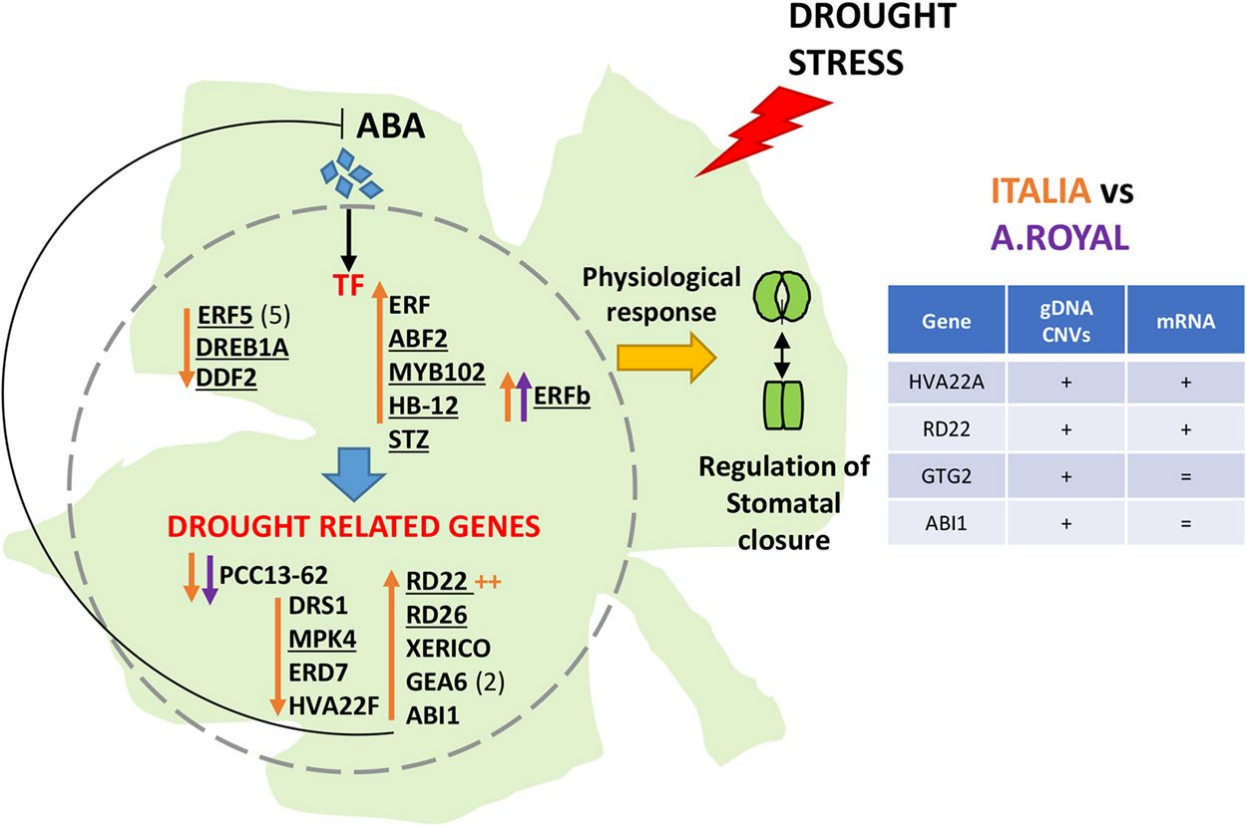
ABA-mediated response to drought stress in cvs. Italia vs Autumn royal. The ABA-responsive transcription factors (TF) belonging to AP2-EREB family: *ERF5* (VIT_16s0013g00980, VIT_16s0013g00990, VIT_16s0013g00950, VIT_16s0013g01060, VIT_16s0013g01050, VIT_16s0013g01030), *DREB1A* (VIT_16s0100g00380) and *DDF2* (VIT_02s0025g04460) are down-regulated under WD in *It*, whereas, ERF (VIT_09s0002g09120), *ABF2* (VIT_18s0001g10450), *MYB102* (VIT_19s0014g03820), the homeobox-leucin zipper protein *HB-12* (VIT_16s0098g01170) and the zing-finger protein *STZ* (VIT_03s0091g00690) are up-regulated. *ERFb* (VIT_09s0002g09140) is the only TF differentially expressed in both *AR* and *It* at WD. TF might regulate the expression of drought related genes, for instance, the down-regulation of desiccation protein *PCC13-62* (VIT_07s0005g00080), *DRS1* (VIT_11s0149g00190), *MPK4* (VIT_15s0046g02000), *ERD7* (VIT_03s0038g02290), *HVA22F* (VIT_12s0142g00440) and the up-regulation of *RD22* (VIT_04s0008g03930), *RD26* (VIT_19s0014g03290), *XERICO* (VIT_12s0057g01330), *GEA6* (VIT_13s0067g01240, VIT_13s0067g01250), *ABI1* (VIT_11s0016g03180). ABI1 proteins might act in a negative feedback regulatory loop of ABA^70^. The table compares copy number variation (CNVs) and mRNA expression of ABA-responsive genes. + indicates higher number of CNVs or mRNA expression in *It* compared to *AR*. The ABA-responsive genes *HVA22A* (VIT_03s0132g00070, VIT_03s0132g00080) and *RD22* showed higher CNs in *It* compared to *AR*, according to their higher expression in *It*, whereas *GTG2* (VIT_07s0005g06120) and *ABI1* showed higher CNs in *It* but did not resulted differentially expressed between cultivars. Genes whose promoters contain ABRE or ABRE-related motifs are underlined. In parentheses it is indicated the number of genes.

A hypothetical scheme of the ABA-mediated mechanisms involved in responses to WD stress in cultivars *AR* and *It* is depicted in Fig. 5. Our results suggest that the increase of ABA and/or of ABA perception in cultivar *It* could be responsible for the transcriptional induction/repression of signalling genes and transcription factors, such as those belonging to AP2/AREB and MYB families. They might affect the transcriptional regulation of drought-related genes. In contrast to the 25 ABA-responsive genes differentially expressed in *It* in response to WD, only two genes resulted differentially expressed in *AR* highlighting that ABA perception is strongly genotype-dependent.

## Conclusion

In conclusion, our analysis confirmed that drought stress response is strictly genotype-dependent and we can infer that the observed responses are dependent on the specific adaptation of each cultivar to environmental conditions. The very limited reprogramming in *AR* suggests it is able to react more rapidly and efficiently than *It* limiting the energy dissipation, and thus confirming a better adaptability.

For the first time, by investigating genomic variants, we were able to identify candidate genes related to genotype-specific response to water deficit in grapevine. We highlighted that structural variants and TEs are some of the primary sources of genomic plasticity, strongly affecting genotype adaptation ability.

Our study represents a step toward the definition of a more rational and efficient use of water resource in viticulture for table grape production.

## Material and Methods

### Plant material, growth and climate conditions

We carried out the study in an experimental vineyard of the Agricultural Research Council, Research Centre for Viticulture and Enology in Turi (Apulia Region) in 2013. The vineyard was located in a trial site on a hilly area (in Turi, southern Italy, long. 40.57 °E, lat. 17.00 °N) at about 190 m a.s.l. Two *Vitis vinifera* L. cultivars, *AR* and *It*, grafted onto ‘140 Ru’ (*V. berlandieri* × *V*. *rupestris*) were used as plant material. Planting density of the vineyard, planted in 2004, was 1600 vines ha^−1^ (2.50 × 2.50 m). Vines were cane pruned (two canes every 12–15 buds per vine) with free-growing shoots (complete overhead canopy separated from fruit). Row orientation was North-South.

The climate is typical Mediterranean semi-arid, characterized by hot dry summers (although short periods of heavy rainfall may occur) and mild rainy winters. The data were collected during the year of the experiment as reported by^30^.

### Irrigation treatments

Two irrigation treatments, based upon a percentage of the net irrigation requirements [NIR = ET_c_ (crop evapotranspiration) – Effective rainfall] from fruit set till harvest, were applied: control full irrigation (FI) and water deficit irrigation (WD) at 100 and 60% of NIR, respectively. For the *It* cultivar an additional point of over-irrigation (OI) was tested, corresponding to an increment of 50% of water supply with respect to FI. ETc was estimated using varying crop coefficients (k_c_) (ET_c_ = ET_o_ × k_c_) based on those proposed by FAO and adjusted for the Mediterranean area and ET_o_ values. ET_o_ was calculated weekly from the mean values of the preceding seven years (2002–2008) using the daily climate data collected in the meteorological station described in the Supplementary note. The applied k_c_ values were 0.35 in April, 0.45 in May, 0.5 in June, 0.75 in July to mid-August, and 0.60 in August to the end of September^61^. The ET_c_ calculated along the season was 270 mm. According to the typical practice adopted in the Apulian region, the vines were drip-irrigated by means of irrigation lines installed 180 cm above the soil surface with drippers spaced 70 cm apart and set to supply water at a constant pressure with two 8 L h^−1^ drippers vine^−1^. Except for the irrigation treatments, all the other standard cultural practices in the vineyard were applied equally to all vines.

### Vine water status and leaf gas exchange

During the steady period of the water potential diurnal curve (generally between 12.30 and 13.30 h), the midday leaf water potential (Ψ_leaf_) was measured seven times during the irrigation period. Measures were made one day before the irrigation application (lowest water availability) and at the mid-cycle, 2–3 days after irrigation (greatest water availability). Two mature exposed leaves per vine (opposite to the cluster, in the middle shoot of the fruit cane) were selected from the canopy, enclosed in plastic bags, and quickly sealed; then, the petioles were cut within 1–2 s and their Ψ_leaf_ was measured immediately in the field by a model 600 pressure chamber instrument (PMS Instrument Company, Albany, USA). The time between leaf excision and chamber pressurization was generally <10–15 s^62^. Gas exchange (leaf photosynthesis rate, stomatal conductance to water vapor, and transpiration rate) was measured on healthy, fully expanded mature leaves exposed to the sun (one leaf on each of 5 vines per treatment), from main shoots located on the exterior canopy (see more details in Supplementary note).

### Fruit quality analyses

Four 7-bunch samples of *AR* and *It* for each treatment, respectively, were randomly harvested at commercial maturity (September 8, 2013) according to a sugar-acid ratio >25. Twenty berries from each bunch were collected, weighed, and their firmness was measured using a deformation tester (Digital Fruit Firmness Tester, Forlì, Italy). Finally, juice was extracted from each sample and used to measure pH, total soluble solids (TSS) as °Brix, and titratable acidity as described in detail in Supplementary note.

Moreover, in the case of *AR* samples, the anthocyanins profile was also determined. Anthocyanins extraction procedure was adapted from the method previously reported^63^ as described in the Supplementary note.

A HPLC-DAD-QqQ (Agilent Technologies, Palo Alto, USA), was adopted for quantitation of tri-hydroxylated anthocyanins, and cyanidin-3-O-glucoside, for quantitation of di-hydroxylated anthocyanins using delphinidin-3-O-glucoside as external calibrations. The detected anthocyanins were summed up and expressed as anthocyanins-3O-glucosides (Ant-glc), anthocyanins-3O-acetyl-glucosides (Ant-ac-glc), anthocyanins-3O-coumaryl-glucosides (Ant-cou-glc), and tri-hydroxylated and di-hydroxylated anthocyanins (Ant-3OH and Ant-2OH) in mg/kg of fresh berries weight (FW).

Three individual vine replicates were assigned to each experimental treatment using a randomized block design. Differences in the quality parameters between the irrigation treatments (FI vs WD) of the two cultivars were tested through pairwaise Student’s t-tests by using STATISTICA 8.0 (StatSoft Inc., Tulxa, OK) package and the statistical tools available in excel.

### Microarray analyses and validation of expression data

Total RNA was extracted from 0.1 g of shoot apex collected at 10% veraison with Total RNA Isolation Mini Kit (Agilent Technologies). RNA integrity was assessed by automated gel electrophoresis on 2100 Bioanalyzer (Agilent Technologies, Amstelveen, Netherlands). cDNA synthesis, labelling and hybridization were performed according to the manufacturer’s instructions (version 6.9.1, Agilent Technologies). Hybridization was carried out on an Agilent custom array. Starting from the assembled *V. vinifera* L. genome sequence (http://www.genoscope.cns.fr/externe/GenomeBrowser/Vitis/), we downloaded annotated transcripts sequences, and using the online tool eArray provided by Agilent Technologies S.p.A., we designed a custom array containing 44 K probes, corresponding to about 26k annotated genes plus EST and transcripts reported in literature as candidate genes for important traits and QTLs available at NCBI databases (www.ncbi.nlm.nih.gov/gene/). The array images were analysed using Agilent Feature Extraction software version 12.0 (Agilent Technologies, Santa Clara, CA).

A selection of seven DEGs was further validated by real time assays. cDNA was prepared as reported in the Supplementary note. Three biological replicates (different plants) were analysed for each sample. All reactions were performed in triplicate. Relative amounts of all mRNAs were calculated using the 2^−ΔΔCt^ method^64^, where ΔCt = Ct(target gene) − Ct(reference gene). The housekeeping gene actin was used as an endogenous reference for normalization.

### Analysis of transcriptomic data

Microarray expression data were processed and analysed using the R package limma (R version 3.1.2, limma version 3.23.2)^65^. Transcripts showing a fold change ≥2 with *p* < 0.05 were considered as differentially expressed. Hierarchical clustering on both entities and conditions was performed using Euclidean distance metric and Ward’s linkage rule.

Overviews of metabolic and regulatory pathways were obtained using MapMan software v3.6.0.

Gene networking analyses were performed using Cytoscape platform v3.4.0^66^ and ClueGo plug-in v2.3.3^67^. The analysis was performed using *Vitis vinifera* as reference. Enrichment/depletion of terms and groups was performed by two-sided hypergeometric test, corrected with Bonferroni step down method. Kappa Score Threshold of 0.4 was applied. Only significant (*p* < 0.005 or *p* < 0.001) pathways belonging to the gene ontology (GO) biological process and Kegg ontologies were considered. GO terms from level 3 to level 8 of GO hierarchy were selected. Kappa Score grouping was applied. Data were visualized as clusters distribution networks. V1 version (http://genomes.cribi.unipd.it/grape/) was used for the annotation of *Vitis vinifera* genes as the most diffused in public databases.

In order to have a comprehensive view on the DEGs belonging to specific pathways of interest, an integrated approach based on multiple annotation methods was used to identify significant genes belonging to target pathways: response to hormone and oxygen-containing compounds, osmotic stress, nitrogen metabolism and photosynthesis. In particular, GO based annotation of Cytoscape was enriched using both *Vitis vinifera* and *Arabidopsis thaliana* as references. In addition, the annotation of MapMan based on GO and enzymatic code (EC) was also included.

The *Vitis vinifera* section of the PlnTFDB - Plant Transcription Factor database^68^ was queried for the correct annotation of the transcription factors differentially expressed in our experiments. Their assignment to specific families was performed after conversion to GSVIVP annotation codes.

### Mining of genomics data

Copy number and single nucleotide variations between *AR* and *It* cultivars were retrieved from data produced by Cardone and co-authors^19^ (76-bp paired-end libraries sequenced using the Illumina GAIIx platform) (Sequence Read Archive, ID: SRP009057).

To calculate and compare the copy number of each region of the grape genome between the two studied cultivars, NGS reads were aligned to the reference using the mrFAST aligner (Alkan *et al*., 2009). The absolute CN of non-overlapping windows of 1 Kbp unmasked sequence (KbUS) was then calculated by using mrCaNaVaR version 0.31. Duplicated and deleted segments were predicted based on 5 KbUS sliding windows^69^: regions with at least five consecutive windows having a CN > 2.5 were identified as segmental duplications, while regions with low read-depth of coverage (CN 1.5 and below) were identified as deletions. Large CNVRs were identified as regions > 10 kbp showing gain or loss using a threshold of L2R > 0.25 for amplifications and L2R < 0.25 for deletions. Here, all CN differences between *AR* and *It* were selected as significant if showing a variation of at least 0.5 copies.

The SNV analysis was performed starting from the data collected in the Supporting Information published by^19^ (SnpEff output). After removing the low-quality calls, we searched for SNVs present in both AR and It when compared to pinot noir reference genome^19^ and showing a different genotype. The genotype is defined as 0/0 for homozygous reference, 0/1 or 1/0 for heterozygous, 1/1 for homozygous alternative.

## Supplementary information

Supplementary Information

Dataset 1

Dataset 2

Dataset 3

Dataset 4

Dataset 5

Dataset 6

Dataset 7

Dataset 8

## Data Availability

All the data produced and described in this manuscript are fully available as Supplementary Datasets. The associated plant material and protocols are fully available in the section Materials and Methods and in the Supplementary note. The gene expression data have been submitted to GEO. The sequencing data used for the genomic analysis are available at SRA under the ID: SRP009057.
