# Supplementary material for: Transcriptomic and genomic structural variation analyses on grape cultivars reveal new insights into the genotype-dependent responses to water stress
Source: Sci Rep. 2019 Feb 26;9:2809. doi: 10.1038/s41598-019-39010-x (PMC6391451; doi:10.1038/s41598-019-39010-x)
Supplement: Supplementary file 1 — Supplementary Information [file 41598_2019_39010_MOESM1_ESM.pdf]

Supplementary Information

Transcriptomic and genomic structural variation analyses on grape cultivars reveal new insights into the genotype-dependent responses to water stress

Catacchio C.R.[2,4], Alagna F.[1,3,4], Perniola R.[1], Bergamini C. [1], Rotunno, S.[1], Calabrese F.M.[2,] Crupi P. [1], Antonacci D.[1], Ventura M.[2]* & Cardone M.F.[1]*

[1]Consiglio per la ricerca in agricoltura e l'analisi dell'economia agraria (CREA), Centro di ricerca Viticoltura ed Enologia, Turi (BA), Italy

[2]Dipartimento di Biologia, Università degli Studi di Bari "Aldo Moro", Bari, Italy

[3]Present affiliation: ENEA, Italian National Agency for New Technologies Energy and Sustainable Economic Development, Trisaia Research Center, Rotondella (MT), Italy

[4]These authors contributed equally to the article

*** Correspondence:**
Maria Francesca Cardone mariafrancesca.cardone@crea.gov.it
Mario Ventura mario.ventura@uniba.it

CONTENTS

Supplementary Dataset Legends

Supplementary note:

1. **Additional methods and results**
   1.1 Leaf gas exchange and fruit quality
   1.2 Anthocyanins analyses
   1.3 Effects of over-irrigation on gene expression
   1.4 Real time analyses
   1.5 MapMan analysis
   1.6 Phytohormone regulation under WD

Supplementary Dataset Legends

Supplementary Dataset 1. Genes up- or down-regulated at water deficit (WD) condition compared to full-irrigation (FI) in Italia and Autumn royal cultivars.

Supplementary Dataset 2. Genes up- or down-regulated in Autumn royal compared to Italia.

Supplementary Dataset 3. Genes up- or down-regulated at water deficit (WD) condition, compared to over-irrigation (OI) in Italia.

Supplementary Dataset 4. Differentially expressed genes belonging to significant pathways.

Supplementary Dataset 5. Copy number differences between Autumn royal and Italia.

Supplementary Dataset 6. Genes showing copy number differences and/or single nucleotide variants and selected for being involved in relevant functional pathways.

Supplementary Dataset 7. SNVs between Autumn royal and Italia causing gain or loss of function.

Supplementary Dataset 8. Differentially expressed genes between Autumn royal and Italia showing copy number differences and/or SNVs causing gain or loss of function.

Supplementary Note

1. Additional methods and results

1.1 Leaf gas exchange and fruit quality

Gas exchange was measured between 09:00 and 11:00 on selected clear days. Measurements were made on healthy, fully expanded mature leaves exposed to the sun (one leaf on each of 5 vines per treatment), from main shoots located on the exterior canopy.

The leaf photosynthesis rate (*A*), stomatal conductance to water vapour (*g*s), and transpiration rate (*E*) were measured with a portable LI-6400 photosynthesis measurement system (LI-COR XT, Lincoln, NE) equipped with a broadleaf chamber. During measurements, leaf chamber temperature was maintained between 30 and 32 °C, leaf to air vapour pressure deficit (VPDl) at 2.0 ± 0.5 kPa, and relative humidity from 40 to 50%. Molar air flow rate inside the leaf chamber was 400 µmol mol^{-1}. All measurements were taken at a reference CO_2 concentration similar to ambient (400 µmol mol^{-1}) and at a saturating photosynthetic photon flux of 1500 µmol m^{-2} s^{-1}, by using a red/blue light source attached to the leaf chamber (Supplementary Table S1).

Supplementary Table S1. Leaf gas exchange and water use efficiency

Sample[a]	Photosynthesis rate A (µmol m^{-2}s^{-1})	Stomatic conductance g_s (mol m^{-2}s^{-1})	Transpiration rate E (mmol m^{-2}s^{-1})	Intrinsic water use efficiency A/gs (µmol mol^{-1})	Instantaneous water use efficiency A/E (µmol mmol^{-1})
It OI	11.06±2.28	0.21±0.04	5.57±0.89	54.28±15.94	2.03±0.50
It FI	9.54±2.65	0.19±0.07	5.47±1.62	56.92±23.14	1.89±0.71
It WD	9.47±2.85	0.15±0.05	4.53±1.45	67.16±19.44	2.22±0.64
AR FI	8.10±1.38	0.12±0.02	3.50±0.47**	69.22±8.56	2.33±0.41
AR WD	7.96±4.57	0.09±0.05	3.16±1.68*	89.7±23.28	2.58±0.70

[a]Parameters measured on Italia (*It*) and Autumn royal (*AR*) plants subjected to different irrigation conditions: over-irrigation (OI), full irrigation (FI), water deficit (WD). Measurements were collected 2-3 days after differential irrigation, corresponding to the first significant variation of Ψ_{leaf}; Data are means ± SD. Statistically significant differences between varieties at the same condition were assessed by *t* test (**p<0.01, *p<0.05).

The juice was centrifuged for 5 min at 4000 g and the supernatant was retained. The pH of undiluted juice of each sample was determined using an equilibrated pH meter (CRISON BASIC 20, Barcelona, Spain). Total soluble solids (TSS) was measured as °Brix using a portable refractometer (Atago PR32, Norfolk, Virginia, USA). Titratable acidity was determined in the juice by titration

with 0.1 N of NaOH (J.T. Baker, Deventer, Holland) to a pH 7 end point with bromothymol blue as indicator and was expressed as gram of tartaric acid L^{-1} (Supplementary Table S2).

Supplementary Table S2. Effects of water deficit on grape production and quality parameters in *It* and *AR*

Condition[a]	N. of bunches	Bunch weight (g)	Vine production (Kg)	Berry fresh weight
It OI	37.10±6.89	826.25±130.39	30.65±0.90	10.01±1.61
It FI	33.40±5.76	857.83±127.68	28.65±0.74	10.37±2.02
It WD	27.30±4.99**	811.75±168.12**	22.16±0.84	9.99±2.49
AR FI	12.60±8.91	2555.20±181.01	32.20±1.61	8.63±0.27
AR WD	13.40±6.80	1485.74±648.80**	19.91±4.41**	5.01±0.20
**Condition**	**TSS (°Brix)[b]**	**pH**	**TTA (g/L)[c]**	**TSS/TTA**
It OI	16.00±0.28	3.47±0.04	5.96±0.21	26.86±1.43
It FI	17.00±0.00	3.49±0.01	5.53±0.29	30.78±1.62
It WD	16.10±0.14	3.49±0.06	4.35±0.11	37.02±0.58
AR FI	15.20±0.40	3.35±0.22	3.93±0.04	38.65±1.41
AR WD	14.03±0.85	2.92±0.28	3.72±0.06	37.74±1.68

[a]Parameters measured on Italia (*It*) and Autumn royal (*AR*) plants subjected to different irrigation conditions: over-irrigation (OI), full irrigation (FI), water deficit (WD); bSST: Total Soluble Solids; cTTA: Total Titratable Acidity. Data are means ± SD. Statistically significant differences comparing OI or WD to FI condition were assessed by *t* test (***p*<0.01).

1.2 Anthocyanins analyses

The skins, manually separated from pulp of frozen 10-berries samples of *AR* (three replicates for each treatment), were rinsed under water to completely separate them from the pulp and dissolve the residual sugar. After that, they were dried with filter paper, weighted, and extracted with 25 mL of 70% methanol containing 1% formic acid in an ultrasonic bath of 130 W and 40 kHz (SONICA 2200 EP, SOLTEC, Milano, Italy); the mixtures, placed in test tubes (50 mL) with screw caps, were sonicated 20 min at room temperature. Subsequently, they were kept in the dark for three days; finally, the extracts were centrifuged at 4000 g for 3 min in an EPPENDORF centrifuge 5810R (Hamburg - Germany), filtered through a 0.45 μm syringe cellulose filter, and analysed by HPLC-DAD-MS/MS.

A HPLC 1100 system, hyphenated with a diode array detector (DAD) and a triple quadrupole QqQ mass detector (Agilent Technologies, Palo Alto, USA), was adopted in this work. 1 μL of extracts were injected onto a Kinetex Evo C18, 5 μm (150 x 2.1 mm i.d.), equipped with a pre-column Security Guard ULTRA cartridge EVO C18 (Phenomenex, Torrance, CA, USA), and analysed following a gradient system with water/formic acid (99:1, v/v) (solvent A) and acetonitrile (solvent B): 0.8 min, 95% A-5%B; 2.12 min, 90% A-10% B; 5.6 min, 88% A-12% B; 8 min, 81% A-19% B; 13.2 min 81% A-19%B; 15.2 min 5% A-95% B; 16.8 min 5% A-95% B; 17.2 min 95% A-5% B. Stop time and post time were set to 17.2 min and 2 min, respectively. The column was kept at 60 °C and the flow was maintained at 0.8 mL min^{-1}. Diode array detection was between 250 and 650 nm,

and absorbance was recorded at 520 nm. Positive electrospray (ESI) mode was used for ionization of molecules with capillary voltage at 4000 V. Nitrogen was used both as drying gas at a flow rate of 12 L min^{-1} and as nebulizing gas at a pressure of 60 psi. Temperature of drying gas was 350 °C. In the full-scan (MS) and product ion (MS/MS) modes, produced by nitrogen collision of ionized compounds in the chosen range at a scan time of 500 ms $cycle^{-1}$, the monitored mass range was from *m/z* 100 to 1200. External calibrations were performed using delphinidin-3-O-glucoside, for quantitation of tri-hydroxylated anthocyanins, and cyanidin-3-O-glucoside, for quantitation of di-hydroxylated anthocyanins. Linearity was checked for delphinidin-3-O-glucoside over the range 1-25 and 25-1000 µg/mL (triplicate injections at five levels, R^2 = 0.9978 and 0.9967, respectively) and for cyanidin-3-O-glucoside over the range 0.2-5 and 5-100 µg/mL (triplicate injections at five levels, R^2 = 0.9988 and 0.9983, respectively). Finally, the detected anthocyanins were summed up and reported as anthocyanins-3O-glucosides (Ant-glc), anthocyanins-3O-acetyl-glucosides (Ant-ac-glc), anthocyanins-3O-coumaryl-glucosides (Ant-cou-glc), and tri-hydroxylated and di-hydroxylated anthocyanins (Ant-3OH and Ant-2OH) in mg/kg of fresh berries weight (FW).

1.3 Effects of over-irrigation on gene expression

Most of the differentially expressed genes (DEGs) between FI and WD are common to those modulated between OI and WD (see Fig. 2 of main text), indicating that under OI these genes follow the same expression trend (up- or down-regulation) of what observed at FI when compared to WD.

Gene networking analyses of the DEGs in the comparison OI-WD revealed a wide modulation of genes involved in primary and secondary metabolism (Supplementary Fig. S1). As observed for the FI-WD comparison, a general down-regulation at WD of genes involved in DNA replication, carbohydrate metabolism, cell wall organization coupled to a modulation (up- or down-) of pathways involved in the responses to stress (osmotic stress, heat, inorganic substances) have been revealed. In addition to what observed in the FI-WD comparison, genes belonging to numerous other pathways were down- or up-regulated. In particular, numerous genes involved in RNA metabolic processes were differentially expressed and a predominant down-regulation has been observed for genes involved in ncRNA metabolic process.

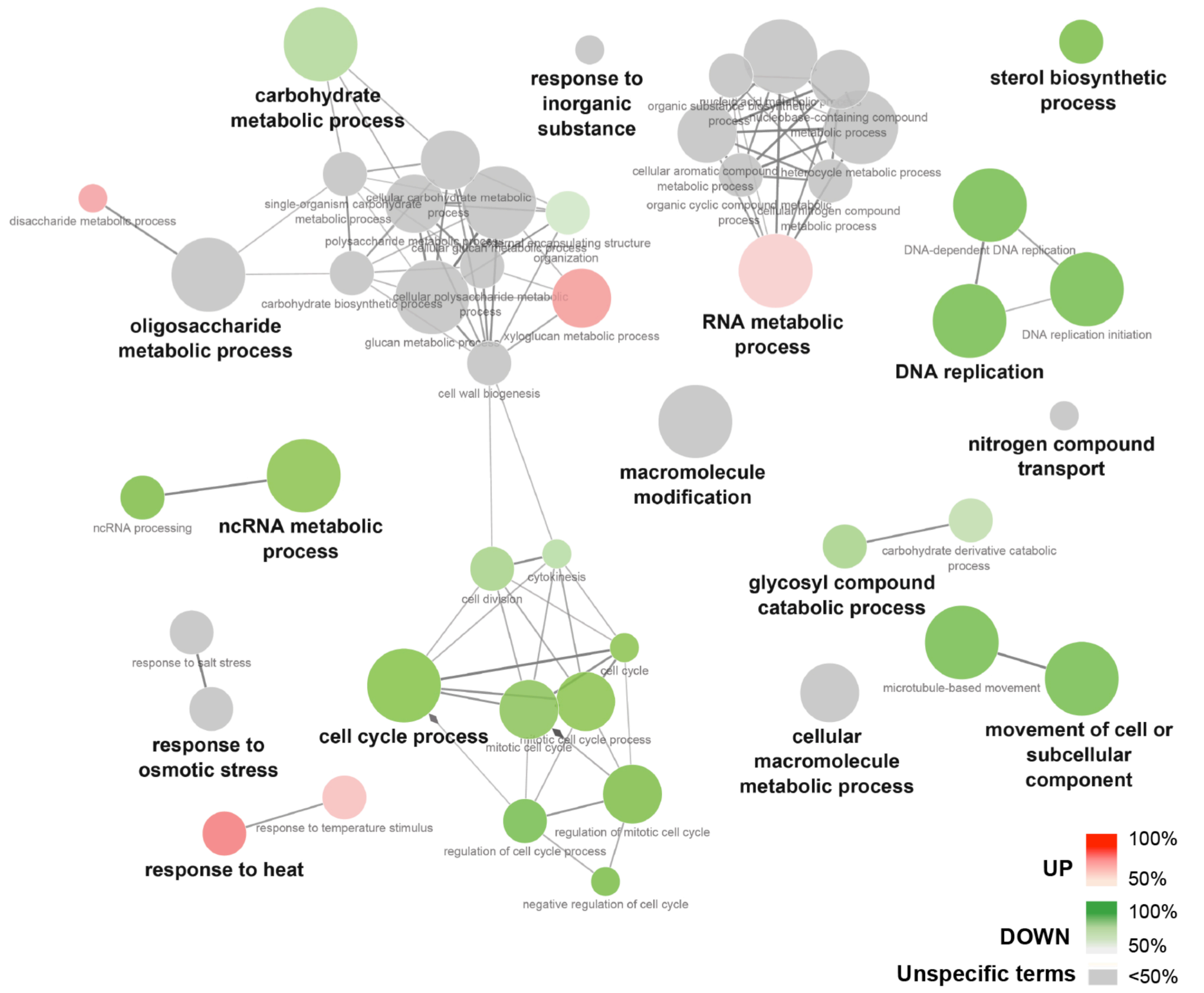

Supplementary Figure S1. Network of differentially expressed genes between over-irrigation and water deficit conditions. Data are visualized as clusters distribution network (Cytoscape, ClueGO App). Only significant (p<0.01) terms belonging to GO biological process ontology were shown. The node size is proportional to the term significance. For Italia cultivar only terms containing at least ten genes were shown. Nodes with up- or down-regulated genes are shown in red or green, respectively. The color gradient shows the proportion of up- and down-regulated genes associated with the term. Equal proportions of both clusters are represented in gray.

1.4 Real time analyses

In order to validate microarray data, we tested by real time analysis: three genes down-regulated under water deficit (*BAN:* VIT_00s0361g00040, *F3H:* VIT_04s0023g03370, *ABI1*: VIT_11s0016g03180); three genes up-regulated under water deficit (*OSM-34*: VIT_02s0025g04340, *ERF*: VIT_09s0002g09140, *DHN*: VIT_04s0023g02480); one gene up-regulated in *It* in OI (*ACD11:* VIT_01s0011g03390) (Supplementary Table S3 and Supplementary Fig. S2).

Supplementary Table S3. Primers used for Real Time analyses.

Gene	V1 annotation	Primer Forward	Primer Reverse	Amplicon length
ACD11	VIT_11s0052g00240	AACCTGTTGTGGGAGGTGTC	GCGTCATGGTTGTCTTCCCT	113
F3H	VIT_04s0023g03370	TGGAGATCGGTGACACAGGA	CATGGCCTCAGACAACACCT	81
BAN	VIT_00s0361g00040	GCACCCCATCGGAAAGAAGA	TGACAGCATAGCCCTTCTGC	98
OSM-34	VIT_02s0025g04340	GTGCCCAGATGCGTACAGTT	GAGGAGGGCTCACATACATGC	200
ABI1	VIT_11s0016g03180	CTTCCGGTTGCACTGTGGTA	AGTTTGAATCCATGGCCAGTGT	145
DHN	VIT_04s0023g02480	GGGGCAGCAACAGAAAGGAA	GACCACGTCCCCTTCATTTCC	90
ERF	VIT_09s0002g09140	TGAGCAGATGATTGAGGAGTTGC	GTATGATGTGGAGAGAAGGCACA	115

Total RNA was extracted from about 0.1g of leaf and tendril, collected at OI, FI and WD conditions, by using Agilent Plant RNA isolation mini kit (Agilent Technologies, Santa Clara, CA, USA) and additionally treated with DNase I amplification grade (Sigma-Aldrich, St. Louis, MO, USA). cDNA was synthesized from 1 μg of total RNA using SuperScript III reverse transcriptase (Invitrogen, Thermo Fisher Scientific, Waltham, MA, USA) according to manufacturer's instructions. Oligo dT and RNaseOUT Recombinant RNase inhibitor (Invitrogen, Thermo Fisher Scientific, Waltham, MA, USA) were used for the first-strand cDNA synthesis. Quantitative Real Time PCR was performed on a PCR Real time Light Cycler 96 (Roche, Basilea, Swizerland) according to manufacturer's protocol using Brilliant III Ultra-Fast SYBR Green qRT-PCR Master Mix (Agilent Technologies, Santa Clara, CA, USA) and gene specific primers (Supplementary Table S4). Primer efficiency was assessed by measuring a standard curve for each gene with six dilution points, each one replicated three times. Only primer pairs that produced the expected amplicon and showed similar PCR efficiency were selected for use. Three biological replicates (different plants) were analysed for each sample. All reactions were performed in triplicate. After each assay, a dissociation kinetics analysis was performed to verify the specificity of the amplification products. Relative amounts of all mRNAs were calculated using the $2^{-\Delta\Delta Ct}$ method (72), where ΔCt = Ct(target gene) - Ct(reference gene). The housekeeping gene actin was used as an endogenous reference for normalization.

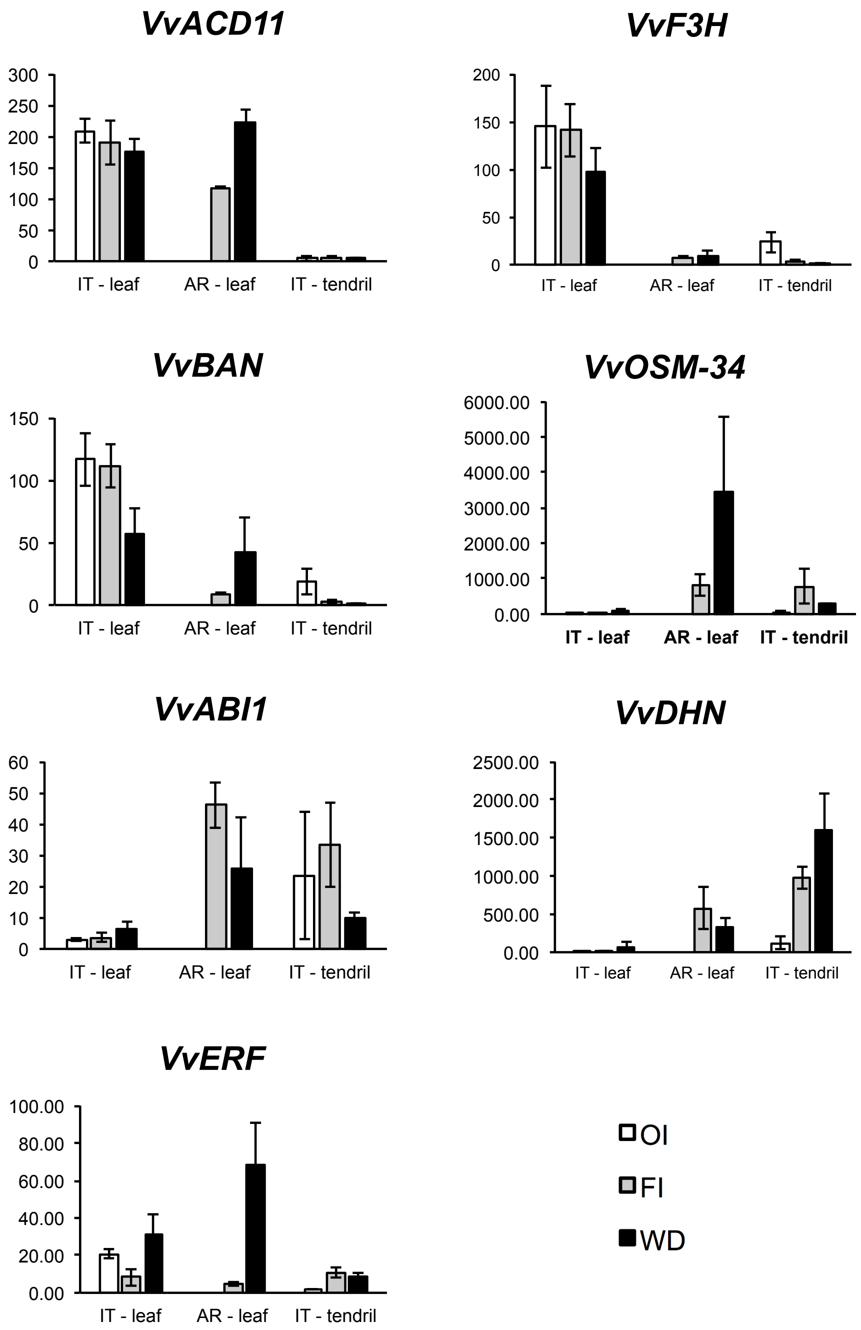

Supplementary Figure S2. Relative mRNA levels of genes putatively involved in the response of grapevine to water deficit stress in leaf and tendril under different water conditions: over-irrigation (white bars), full-irrigation (gray bars) and water deficit (black bars).

1.5 MapMan analysis

Microarrays expression data were properly rearranged customized as input files and mapped on phytozome v9.0 annotation dataset of *Vitis vinifera* downloaded from MapMan store (mapman.gabipd.org/mapmanstore). MapMan analysis provided an overview of the metabolic pathways and regulatory networks affected by water deficit in the two studied cultivars (Supplementary Fig. S3).

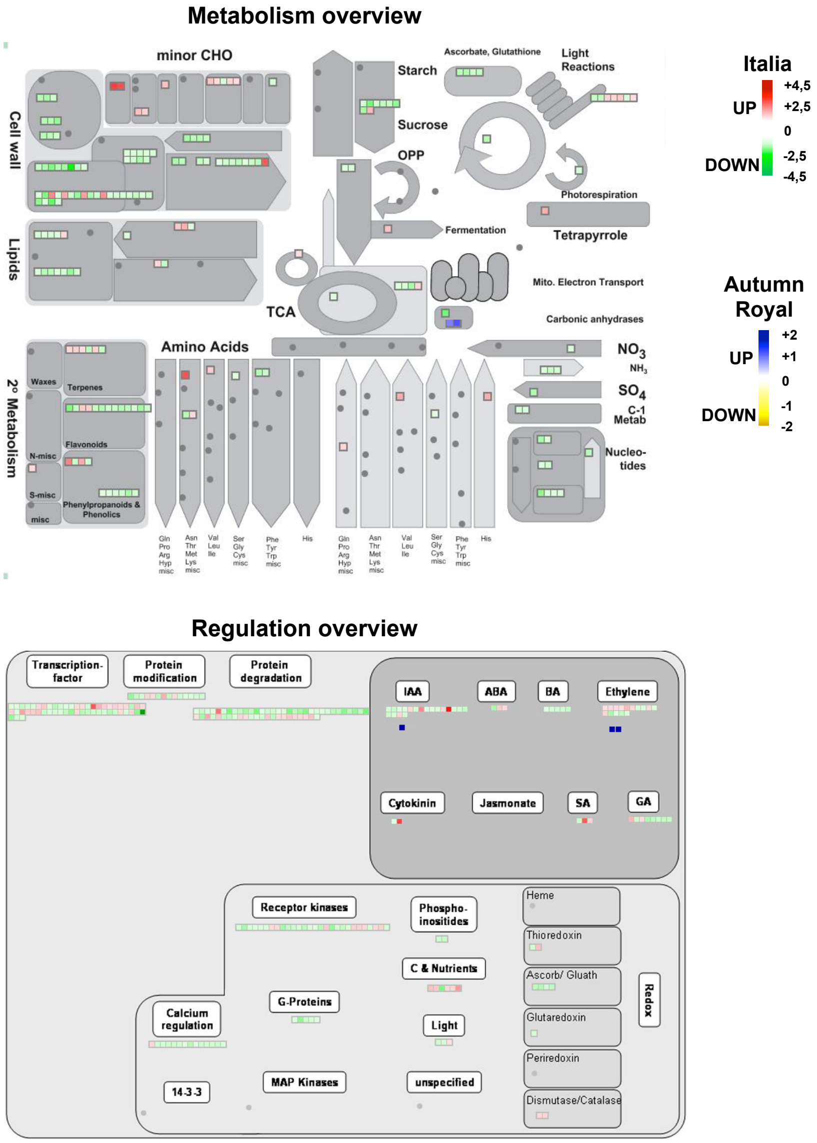

Supplementary Figure S3. MapMan metabolic and regulatory overview maps showing differences in the transcript levels of grapevine genes at water deficit (WD) compared to full irrigation (FI). Maps were constructed with MapMan software v3.6.0. Red and green squares represent, respectively,

genes up- or down-regulated at WD compared to FI in Italia cultivar, whereas, blue and yellow represent those up- or down-regulated, respectively, in Autumn royal.

The DEGs of *It* involved in the main pathways affecting photosynthesis (light reactions, calvin cycle and photorespiration) were grouped and visualized in the MapMan maps based of their specific functions (Supplementary Fig. S4). No DEGs affecting photosynthesis were identified in *AR*.

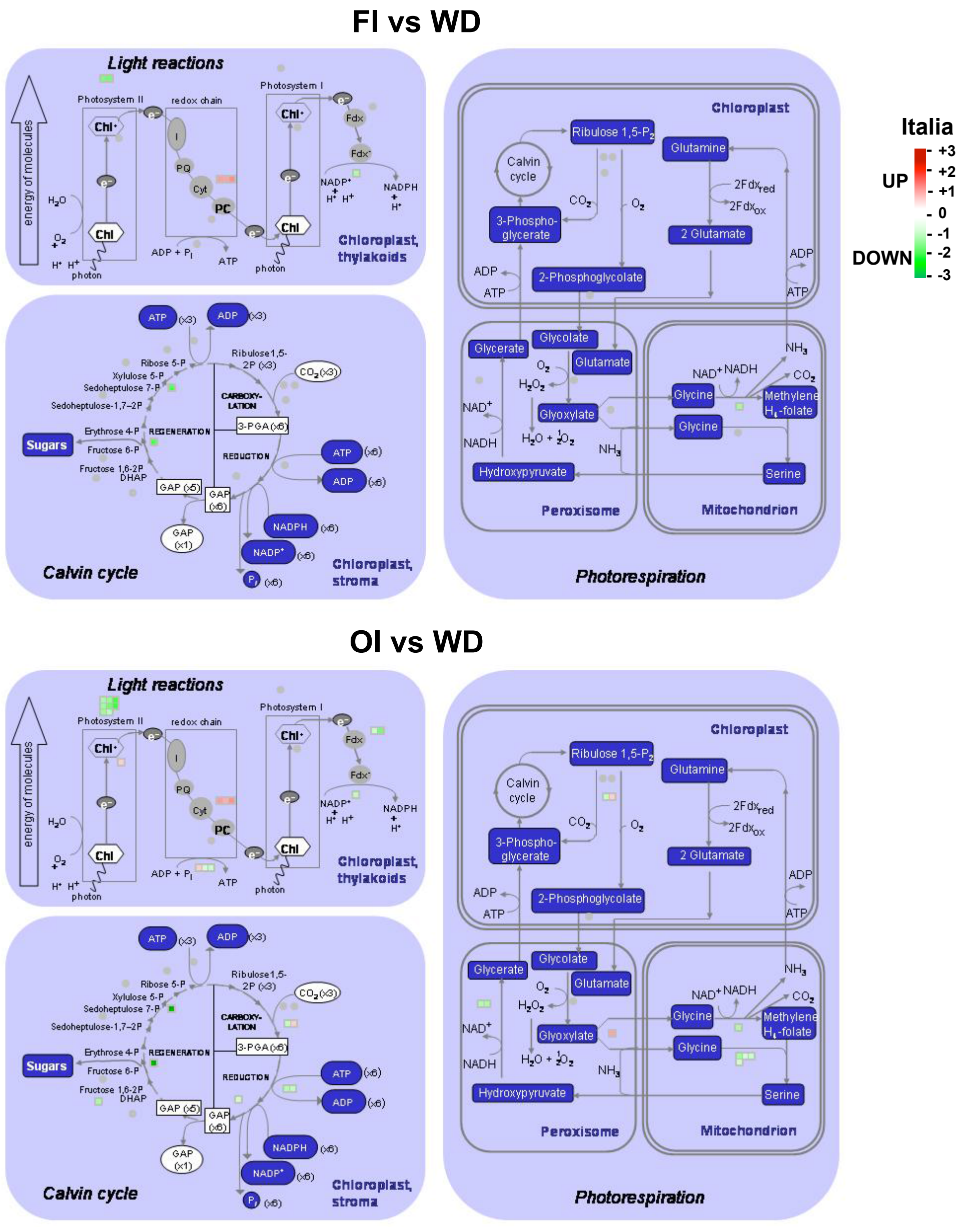

Supplementary Figure S4. MapMan visualization of photosynthesis showing differences in the transcript levels of grapevine genes at water deficit compared to full irrigation (above) or at water deficit compared to over-irrigation (below), in Italia cultivar. Maps were constructed with MapMan software v3.6.0. Red and green squares represent genes up- or down-regulated, respectively.

1.6 Phytohormone regulation under WD

According to the important role of phytohormones in the regulation of plant stress response we found DEGs involved in the perception, signalling and metabolism of the following phytohormones: abscisic acid (ABA), auxin (AUX), ethylene (ET), jasmonic acid (JA), gibberellin (GA), cytokinin (CK), salicylic acid (SA) and brassinosteroid (BR) (Supplementary Table S4). Considering the high number of ABA-responsive genes among the genes differentially expressed under WD and the pivotal role of this hormone in the modulation of drought response in plants, we searched for ABA response elements (ABRE) in the promoter region (1000 nt upstream the start codon) of hormone-responsive genes. As expected, we found ABRE-related motifs in most of the ABA-responsive genes and in numerous other hormone-responsive genes (Supplementary Table S4). Interestingly, differences between *AR* and *It* were identified. These results are in accordance to the genotypic-specific response to water deficit stress.

Supplementary Table S4. Hormone-responsive genes differentially expressed under water deficit

Hormone	N. of genes	Gene name	
		DOWN	UP
ABA	25	DRS1 (VIT_11s0149g00190), HVA22F (VIT_12s0142g00440), PCC13-62** (VIT_07s0005g00080), **ERF5** (VIT_16s0013g00980, VIT_16s0013g00990, VIT_16s0013g00950, VIT_16s0013g01060, VIT_16s0013g01050, VIT_16s0013g01030), **DREB1A** (VIT_16s0100g00380), **DDF2** (VIT_02s0025g04460), **MPK4** (VIT_15s0046g02000), ERD7 (VIT_03s0038g02290)	ABI1 (VIT_11s0016g03180), GEA6 (VIT_13s0067g01240, **VIT_13s0067g01250**), VIT_201s0010g01840, **MYB102** (VIT_19s0014g03820), XERICO (VIT_12s0057g01330), **ERF**** (VIT_09s0002g09140), **HB-12** (VIT_16s0098g01170), **STZ** (VIT_03s0091g00690), **ABF2** (VIT_18s0001g10450), **RD22** (VIT_04s0008g03930), **RD26** (VIT_19s0014g03290)
AUX	18	VIT_03s0038g01080, VIT_03s0038g01100, VIT_03s0038g01120, VIT_03s0038g01160, VIT_03s0038g01180, VIT_03s0038g01220, VIT_03s0038g01260, **VIT_04s0023g00520**, **VIT_04s0023g00540**, **VIT_10s0116g01020**,	VIT_10s0597g00010, **AILP1** (VIT_05s0020g02770), **VIT_19s0014g03130**

		VIT_18s0001g03610 ATAUX2-11 (VIT_07s0141g00270), PAP2 (VIT_09s0002g04080), **IAA19** (VIT_09s0002g05150, **PIN5** (VIT_04s0023g00320)	
ET	9	ERF5 (VIT_16s0013g01070, VIT_16s0013g00970)	**DMR6** (VIT_16s0098g00860), **VIT_08s0007g03040**, VIT_06s0004g06790, **VIT_08s0007g03050**, **VIT_03s0017g00830**, **VIT_03s0017g00710**, ERF (VIT_09s0002g09120)
BR	5	CYP51G1 (VIT_01s0150g00210), **DWF1** (VIT_01s0010g01200), HYD1 (VIT_06s0004g02100), SMT1 (**VIT_15s0048g01430**, VIT_13s0064g00440)	
JA	5	JAZ8 (VIT_04s0008g00110, **VIT_10s0003g03790**, **VIT_10s0003g03800**), FNR2 (VIT_18s0001g14450), **JMT** (VIT_04s0023g03810)	
CK	2	WOL (VIT_01s0011g06190)	HK3 (VIT_17s0000g04920)
SA	6	**CPK29** (VIT_18s0001g00990)	**PR1*** (VIT_03s0088g00810, VIT_03s0088g00890), **PRB1*** (VIT_03s0088g00690), VIT_01s0011g05930, VIT_01s0011g05940
GA	6	GASA4 (**VIT_14s0108g00740**, VIT_00s0189g00070, VIT_00s0189g00060), **VIT_17s0000g06210**	**GID1B** (VIT_07s0104g00930), **VIT_01s0026g00620**
General hormone response	4		STH2 (VIT_03s0038g00340), **VIT_03s0038g04390,** VIT_09s0002g03610, VIT_16s0050g00390,

Genes involved in hormone perception, signalling and metabolism have been indicated. Down- and up-regulation is referred to *It* cultivar except for those differentially expressed exclusively in *AR* (*) and those differentially expressed in both *It* and *AR* (**). Genes whose promoters contain ABRE or ABRE-related motifs are indicated in bold. ABA: Abscisic acid; AUX: Auxin, ET: Ethylene; BR: Brassinosteroid; CK: Cytokinin; SA: Salicylic Acid; GA: Giberrelic acid.
